# The Effects of Carbohydrates, in Isolation and Combined with Caffeine, on Cognitive Performance and Mood—Current Evidence and Future Directions

**DOI:** 10.3390/nu10020192

**Published:** 2018-02-09

**Authors:** Neil Bernard Boyle, Clare Louise Lawton, Louise Dye

**Affiliations:** Leeds Nutrition and Behaviour Group, School of Psychology, University of Leeds, Leeds LS2 9JT, UK; c.l.lawton@leeds.ac.uk (C.L.L.); l.dye@leeds.ac.uk (L.D.)

**Keywords:** carbohydrate, caffeine, cognitive performance, subjective mood, glycemic response, glucose

## Abstract

This review examines the effects of carbohydrates, delivered individually and in combination with caffeine, on a range of cognitive domains and subjective mood. There is evidence for beneficial effects of glucose at a dose of 25 g on episodic memory, but exploration of dose effects has not been systematic and the effects on other cognitive domains is not known. Factors contributing to the differential sensitivity to glucose facilitation include age, task difficulty/demand, task domain, and glucoregulatory control. There is modest evidence to suggest modulating glycemic response may impact cognitive function. The evidence presented in this review identifies dose ranges of glucose and caffeine which improve cognition, but fails to find convincing consistent synergistic effects of combining caffeine and glucose. Whilst combining glucose and caffeine has been shown to facilitate cognitive performance and mood compared to placebo or glucose alone, the relative contribution of caffeine and glucose to the observed effects is difficult to ascertain, due to the paucity of studies that have appropriately compared the effects of these ingredients combined and in isolation. This review identifies a number of methodological challenges which need to be considered in the design of future hypothesis driven research in this area.

## 1. Introduction

The potential facilitative effects of carbohydrates (CHOs) on cognitive performance were first proposed in the 1950s [1]. Since then the capacity of CHO intake to enhance cognitive performance, or attenuate cognitive impairment, has been widely examined. A rise in the popularity of “energy” drinks that combine CHOs with caffeine, and claim to offer beneficial performance effects, has resulted in a growing literature examining the cognitive effects of combining CHO with caffeine. This review outlines the existing evidence of the capacity of CHOs in isolation and combined with caffeine to offer facilitative cognitive performance effects. Evidence of the effects these ingredients on measures of subjective mood will also be examined.

Table 1 summarizes the cognitive domains commonly employed in studies that have examined the effects of CHO intake on cognitive performance. Tests of cognitive function measure a range of cognitive modalities, including memory, attention and vigilance, information processing, and accuracy and speed of response [2]. These tasks tend to measure components of performance that may tap into more complex skills; for example, psychomotor skill may be a proxy measure of driving performance [3].

## 2. Carbohydrates and Cognitive Function

### 2.1. Glucose

Glucose is the primary monosaccharide in mammalian metabolism and most abundant dietary sugar-accounting for ~80% of the end product of CHO digestion [4]. Glucose is virtually the sole fuel for the brain except during prolonged starvation when liver ketone bodies are oxidized. Due to the inability to store fuel the brain requires a continual supply of glucose, an estimated 120 g per day [5]. Glucose is by far the most systematically examined CHO in relation to the moderation of cognitive function, and forms a prototypical research model of the nutrition–behavior axis. The facilitative effects of glucose on cognitive performance have been investigated in diverse populations (e.g., adolescents [6]), young adults [7,8], older adults [9] and individuals with cognitive impairments [10] and dementia [11]).

To date, examination of the effects of glucose facilitation has predominantly focused upon episodic memory. Table 2 highlights that the most consistent effects of glucose have been demonstrated in this cognitive domain. Evidence of the facilitation of episodic memory suggests a specific enhancing effect of glucose intake on cognitive domains associated with the function of the hippocampus. In support of this, facilitative effects have also been reported for additional hippocampal-dependent cognitive functions: recognition memory [12,13,14,15,16]; visuospatial memory [17,18] and visuospatial functioning [19,20]. 

However, glucose enhancement of cognitive domains that are not closely associated with hippocampal function has also been demonstrated. For example, processing speed and reaction time [21], working memory [13,22,23]; problem solving [24] and attention [25,26,27,28] have all been shown to be sensitive to an acute glucose load.

### 2.2. Factors Moderating the Effect of Glucose

Examination of the study outcomes shown in Table 2 shows that whilst the enhancement of episodic memory has been the most consistently reported outcome, no effect of acute glucose intake is often reported, even when comparable doses and cognitive tests are employed (e.g., [29,30,31,32]). This inconsistency in the facilitative effect of glucose administration is evident both across and within cognitive domains. Such heterogeneity in the evidence suggests a role for additional factors mediating the relationship between glucose and cognitive function.

#### 2.2.1. The Effect of Dose

The majority of studies have administered an acute 25 g glucose dose. This is often cited as the optimum dosage for the facilitative effect of glucose on memory [9]. This dose also provokes a human blood glucose increase commensurate with the blood glucose levels shown to have facilitative cognitive effects in rats (100 mg/kg [67]). An inverted U-shaped dose response curve between glucose dose and memory performance has been demonstrated in animal models [68,69,70]. There is also evidence to suggest this relationship may be bimodal with performance peaks at 100 mg/kg and 2000 mg/kg [71]. Evidence from clinical populations (e.g., diabetic samples) demonstrate impaired performance associated with hypo- and hyperglycemia [72]. Support for an inverted U-shape relationship has been demonstrated in elderly humans [73]. A limited number of studies have systematically examined the dose response relationship in young healthy samples. Azari et al. [29] found no effects of 0, 30 or 100 g of glucose on episodic or recognition memory (*M_age_ =* 21 years). Meikle et al. [26] administered 0, 25 and 50 g of glucose to young (*M_age_ =* 21.8 years) and middle-aged (*M_age_ =* 38.4 years) samples. Episodic memory was generally enhanced by glucose intake (25 g and 50 g) with evidence of greater facilitation of performance in the middle-aged sample. Messier et al. [37] administered a broader range of acute doses (0, 100, 300, 500, 800, and 1000 mg/kg of body weight) to examine the dose-response curve of the effect of glucose on episodic memory (*M_age_ =* 21.3 years). The 300 mg/kg and 800 mg/kg doses resulted in attenuation of the commonly observed decline in the primacy effect (enhanced recall of information presented first) as respondents learnt an increasing number of word lists. This suggests a bimodal relationship between glucose dose and facilitation (10 mg/kg, 100 mg/kg, 500 mg/kg and 1000 mg/kg did not facilitate performance). Sünram-Lea et al. [16] examined episodic memory recall and recognition and working memory after administration of 15, 25, 50, and 60 g glucose loads (*M_age_ =* 20 years). Facilitation of spatial working memory, immediate and delayed recall, and recognition were reported for the 25 g glucose dose. No facilitative effects were demonstrated at lower (15 g) or higher (50 g and 60 g) doses. This supports the proposition of a specific optimal glucose dose of 25 g. However, divergent dose response curves were evident dependent upon cognitive domain. An inverted U-shape dose response profile was largely demonstrated for episodic memory performance. However, performance did not fall below control levels at high doses as would be predicted by an inverted U curve. The dose-response relationship of working memory performance adhered to a cubic trend characterized by facilitation at the lowest and highest doses. Spatial working memory enhancement was significant at 25 g but additional enhancement trends were observed at higher doses, suggestive of a quartic trend [16].

The current data suggests the facilitative glucose dose–response relationship is complex and may be domain specific. Whilst there is some support for the proposition that 25 g is optimal for facilitative effects on memory performance, this evidence is primarily representative of the enhancing effects on episodic memory; specifically, delayed, verbal episodic memory [74]. However, a number of studies have failed to demonstrate facilitative effects of a 25 g dose. Enhanced performance has also been demonstrated after lower (15 g) and higher (50, 60 and 75 g) doses. If the facilitative effect of glucose followed an inverted U-shaped dose response curve, impaired performance should be demonstrated at very low and high doses. There is little evidence to support this proposition in the limited number of dose response studies that have been undertaken in healthy young samples. Flint and Turek [46] reported impaired attention performance after 100 mg/kg glucose drink. However, 500 mg/kg did not impair performance. This finding contradicts impairment as a function of increasing dose.

#### 2.2.2. The Effect of Age

Reduced glucose control [44,75] and dysregulation of neuroendocrine processes associated with cognitive function and glucose regulation (e.g., adrenaline [76]) are common corollaries of ageing. Cognitive capacity also diminishes as a function of age resulting in a tendency for poorer performance on cognitive tasks in older vs. younger adults [77]. The combination of a compromised glucoregulatory system and deficits in cognitive function may result in an increased sensitivity to the facilitative effects of glucose in older adults. Indeed, differential effects of glucose administration in older samples are evident. Hall et al. [22] reported greater enhanced episodic memory after 50 g of glucose in elderly (*M_age_* = 67.4) vs. young (*M_age_* = 20) adults. Working memory performance was also selectively enhanced only in the young. Further, individual glucose tolerance predicted memory performance in the elderly only (effects of glucoregulatory control discussed in Section 2.2.4).

Meikle et al. [26] highlighted the importance of task demand on the relationship between glucose facilitation and age. The level of task demand moderated the degree of glucose enhancement of short-term episodic memory in middle-aged (*M_age_* = 38.4) vs. young (*M_age_* = 21.8) adults. Glucose intake (25 g and 50 g) restored middle-aged adults’ memory performance to that of their young counterparts only on higher cognitive load trials. This selective facilitative effect may be indicative of the capacity of glucose to offer greater benefit to those that are not performing close to ceiling. Young, healthy adults may be operating near the limit of cognitive capacity, leaving little room for performance improvement. Conversely, age-related cognitive decline in middle-aged adults may result in cognitive deficits under higher cognitive loads which may be sensitive to glucose facilitation.

#### 2.2.3. The Effect of Task Demand

A number of studies have failed to demonstrate glucose enhancement in healthy young adults when episodic memory was assessed under single task conditions (e.g., [29,31,33,57]). Studies that do report facilitative effects under single task conditions often demonstrate primacy and recency effects [35,37]. Tasks that place a high demand on cognitive resources, or performance assessed under dual/multi-task demands, appear more sensitive to the facilitative effects of glucose (e.g., [7,15,27,35,36,48]). These studies suggest glucose may preferentially facilitate tasks that require a high cognitive processing load.

The dual task paradigm (the performance of two concurrent or consecutive tasks to increase distraction or cognitive load) has been commonly employed to demonstrate the effects of cognitive load on the relationship between glucose and cognitive performance. For example, Sünram-Lea et al. [15] reported episodic memory enhancement only when participants were concurrently performing an additional task. Similar glucose enhancement under conditions of divided attention have been reported [7,48,55]. The level of task demand also appears to moderate the glucose facilitation effect. Cognitive tasks that are more cognitively demanding may be particularly sensitive to glucose loading. Brown and Riby [63] demonstrated glucose facilitation only for the most demanding episodic memory and attention task conditions. Glucose results in greater performance enhancement on incongruent, thus more difficult, trials in the Stroop task paradigm [61]. Preferential enhancement of recall of low imagery word pairs and longer words lists has also been reported [47]. Related to increased cognitive demand, Reay et al. [27] suggest the facilitative effect of glucose may only appear as fatigue increases when faced with demanding, prolonged tasks.

The mediating role of task demand and load is underpinned by the assumption that cognitive capacity and/or glucose resources are ‘depleted’ by the excessive demands placed upon them. The energy requirements of the brain are substantial, approximately 20%–30% of an organism’s basal metabolic output [78]. The brain has long been considered to lack storage capacity for energy substrates and is therefore reliant upon the aerobic degradation of glucose and oxygen supplied in the bloodstream [43]. Some have argued that the metabolic energy cost of effortful, controlled or executive cognitive processes are higher than the cost of automatic or reflexive processes [50]. Therefore, cognitively demanding tasks may consume more glucose and may be more sensitive to manipulations of peripheral blood glucose. Animal models have demonstrated selective reduction of extracellular glucose concentration in the hippocampus mediated by the level of cognitive demand [79]. There is limited evidence of lowered peripheral glucose levels associated with performance on demanding cognitive tasks in humans [43,80,81]. Authors have inferred a directional effect, assuming that that more demand leads to lower peripheral glucose.

The cognitive act of self-control is one cognitive domain that has been proposed to demonstrate the specific effects of depleted cognitive capacity at high demands, and the direct restorative effects of glucose intake. Acts of self-control require the effortful inhibition of predominant responses, emotions, thoughts, and impulses, permitting behavior to vary adaptively moment to moment [82,83]. The strength model of self-control asserts that self-control is a uniquely demanding domain of cognition, and self-control tasks deplete a limited cognitive resource resulting in reduced subsequent self-control performance; a state of ‘ego-depletion’. Gailliot and Baumeister [50] proposed that glucose is the direct central energy source of self-control. This proposition was founded on evidence of: (i) reduced blood glucose levels after initial exertion of self-control; (ii) an association between subsequent, post-depletion, self-control performance and blood glucose decline; and (iii) attenuation of the detrimental ego depletion effect on self-control performance after ingestion of glucose, but not artificial sweetener [81].

The capacity of glucose ingestion to counteract the impairing effect of ego depletion has been demonstrated (e.g., [51,52,81]). However, these studies provide scant information as regards glucose dose, sample composition, and the methods of depleting and measuring self-control performance often appear arbitrary (e.g., writing about one’s death [81]). Moreover, the precise role of glucose in self-control performance remains indistinct. Firstly, a number of studies have demonstrated that glucose can influence performance on self-control tasks in a non-energetic manner. Merely sensing carbohydrates, but not artificial sweeteners, in the oral cavity can confer a restorative benefit on cognitive self-control performance under conditions of ego-depletion [84,85,86]. The positive effect of carbohydrate oral rinsing has also been demonstrated in physical endurance performance [87,88], conferring greater performance benefits than ingestion [89]. Such findings suggest a potential motivational rather than metabolic effect of carbohydrates on performance, underpinned by activation of motivational neural reward pathways [88,90,91].

It is important to note that evidence of lowered peripheral blood glucose related to the level of cognitive demand is weak. Fairclough and Houston [80] and Scholey et al. [43] reported a peripheral decrease in capillary blood glucose <1 mmol/L. Both studies employed commercially available fingerprick based capillary blood glucose analyzer devices to measure glucose levels in a healthy sample. These devices are not designed to accurately detect blood glucose excursions outside the euglycemic range. Such effects should therefore be treated with caution. Subsequent attempts to replicate the moderation of peripheral blood glucose by exertion of self-control have also not supported the finding that demanding tasks consume more glucose [91]. Indeed, our laboratory recently failed to find any moderation of capillary blood or interstitial glucose by self-control exertion, rigorously assessed using formal laboratory standard capillary blood glucose analysis techniques and continuous interstitial glucose monitoring [92].

Regulation of glucose transport across the blood brain barrier (BBB) occurs via GLUT1 transporters but this process is not well understood [93]. Glucose levels in the brain are approximately 30% of those in peripheral blood [94]. Long term elevations in peripheral glucose result in decreased glucose transport across the BBB [95]. During brain activation, utilization and local concentrations of glucose have been shown to alter. An increase in glucose uptake by the brain in young males undertaking a complex visuo-spatial motor task was observed in a PET study [96], and in rats, a decrease in hippocampal interstitial glucose levels proportional to the difficulty of the maze was observed [79]. However, in both studies peripheral glucose concentrations remained unchanged. This suggests that cognitive demand will be accompanied by increased local glucose metabolism in those brain areas engaged in specific tasks. Moreover, the amount of glucose required for acts of self-control and cognitively demanding tasks is likely to be negligible in absolute brain energy cost terms. Furthermore, reduced peripheral glucose by cognitive demand is unlikely considering the efficiency of homeostatic systems in maintaining brain energy levels [85]. Behavioral evidence for an effect of task demand is also mixed. Facilitative glucose effects on lower (serial 3’s), but not higher (serial 7’s) demand tasks [13], and no effects of dual task demand [66] have been demonstrated. This is counter to what would be expected if glucose uptake changed in response to demand.

#### 2.2.4. The Effect of Glucoregulatory Control

Glucose regulation appears to be a key moderator of optimal cognition functioning. Hypoglycemia, induced experimentally, or in type 1 diabetes, is associated with impaired cognitive performance [97,98]. Further, poor glycemic control in type 2 diabetes is associated with impaired memory [99], and increased risk of cognitive decline [100]. Impaired glucose tolerance (IGT), which is associated with insulin insensitivity and is increasingly prevalent in the general population due to the increased incidence of obesity, also affects cognitive function [101]. Intranasal insulin and thiazolidinediones (which improve insulin sensitivity) improve memory function. This effect is linked to lowered blood glucose concentrations rather than altered insulin levels [102].

The literature suggests that the facilitative effects of glucose on cognitive performance may be moderated by an individual’s ability to regulate their blood glucose response. Therefore, whilst it is commonly stated that a 25 g glucose dose is optimal for facilitative effects, the failure of the majority of studies to take into account the mediating effects of glucoregulatory control, and factors associated with the regulation of glucose (e.g., age, weight, BMI), may account for some heterogeneity in the evidence.

The variable effects of glucose regulation have been shown as a function of glucoregulatory control and age. For example, performance deficits in an elderly sample demonstrated after intake of 50 g of glucose were partly moderated by differences in glucose regulation [73]. Craft et al. [34] demonstrated that performance on a verbal episodic memory task was differentially affected dependent upon glucose regulation in elderly (*M_age_* = 68.5) vs. young (*M_age_* = 20.8) adults. Elderly performance was enhanced in good, and impaired in poor, glucose regulators (indexed by degree to which blood glucose returned to baseline levels). Conversely, younger adults showed the opposite response pattern: prolonged elevated blood glucose levels were associated with enhanced performance and good regulatory control was associated with impaired performance.

The effects of glucoregulation in studies of exclusively young, healthy samples are mixed, with evidence of selective effects in individuals with poor or good glucoregulatory control. Evidence of selective facilitative effects in those with poor regulatory control [13,38], but no effects [44], or impairment [57] in those with better regulatory control has been reported. Conversely, individuals with better glucoregulatory control have been shown to be particularly sensitive to the facilitative effects of glucose (e.g., [26]). Sünram-Lea et al. [16] also reported tentative (due to doubts about the methodology adopted to classify poor and good glucose regulation) facilitative effects of higher glucose loads in good glucose regulators. This study also highlighted the moderating effect of weight and body composition. The data suggested that individuals with low and medium BMI (<25 kg/m^2^) show facilitative effects of high acute glucose loads, whilst higher BMI (>25 kg/m^2^) was associated with performance decrements. Poorer glucose regulation is demonstrated in the overweight and obese, however, no direct evidence of BMI moderating glycemic response to a glucose load was reported in this study. Body mass index was positively associated with basal fasted glucose levels suggesting this effect may be mediated by the long-term action of insulin resistance more evident in overweight and obese individuals.

#### 2.2.5. Emotional Valence

Emotionally laden stimuli (e.g., words, pictures) are more memorable than neutral stimuli; the ‘emotional enhancement effect’ [103]. This effect has been demonstrated across a number of cognitive domains, but predominantly recognition and recall. The effect is likely underpinned by the acute emotional arousal activating the release of glucocorticoids and adrenaline. A major physiological role of both of these hormones is to temporarily increase energy production, specifically the provision of increased metabolic fuel via increased glucose availability [58]. There is some modest evidence that exposure to emotionally valenced words can raise plasma glucose levels [104,105]. This suggests memory for emotionally valenced stimuli may be particularly sensitive to acute glucose manipulations. A number of studies have examined the potential for glucose to moderate the emotional enhancement effect. However, the findings to date are mixed. Both 50 g and 100 mg/kg impaired emotionally valenced spatial memory performance [19]; 50 g glucose enhanced performance for neutral trials. Further studies have demonstrated no additional effect above the standard emotional enhancement effect of 25 g of glucose [45,49]. Brandt et al. [58] proposed the mixed findings may be a dosing problem. Whilst 25–50 g may be sufficient for the enhancement of neutral stimuli, commonly adopted in studies of episodic memory, a lower dose may be optimal for the enhancement of emotional stimuli as blood glucose levels may already have been augmented by mere exposure to the arousing stimuli. However, only a marginal effect of a 15 g glucose dose on recognition memory was observed. This suggests glucose administration does not affect the memory advantage evident for emotional stimuli. It is likely that an independent relationship exists between blood glucose levels and memory of emotional material.

#### 2.2.6. Expectancy Effects

The capacity of merely sensing glucose in the oral cavity to enhance cognitive performance raises the possibility of potential non-metabolic facilitative effects of glucose. Support for this proposition comes from studies demonstrating the crucial moderating factor of the expectancy of consuming glucose. Expectations relating to the effects of caffeine and alcohol intake have been shown to moderate cognitive performance [106]. Similar effects may be expected for the consumption of glucose. Indeed, comparing participants who were correctly or incorrectly informed of the content of a drink, Green et al. [41] demonstrated improved vigilance performance only when respondents were given a drink congruent message (i.e., glucose intake with expectancy of intake). However, Stollery and Christian [28] suggest the effects of expectancy beliefs for glucose may be modest and relatively isolated to internal indices of specific cognitive tasks. For example, inducing within-task trade-offs, for example, recall of more high imageability words vs. low imageability words if expecting glucose, without any tangible effect on overall performance (i.e., number of words recalled). Therefore, the authors suggest expectancy effects are unlikely to be confused with glucose enhancement effects. However, the potential for expectancy effects to augment specific domains of performance [41], or change the nature of performance within specific domains [28], suggests data on participant expectations should be collected.

A related effect is the mediating impact of thirst on glucose facilitation. Scholey et al. [56] reported participants who self-reported being less thirsty at baseline recalled significantly more, and those thirstier significantly fewer, words after glucose intake vs placebo. However, no further attempt has been made to corroborate this finding. The potential mediating roles of subjective expectancy and thirst on the enhancing potential of glucose are worthy of further examination.

### 2.3. Glucose and Subjective Mood

There is increasing interest in the capacity for glucose to enhance subjective mood. A number of studies examining the effects of glucose on cognitive performance additionally measured participants’ subjective ratings of alertness, energy, and fatigue. Such measures were considered to index the perceived level of arousal following glucose intake. Recently, ‘mental energy’ has been proposed as a construct that can be employed to define the facilitative effects of macronutrient intervention on subjective arousal [107]. Mental energy is defined “as the ability to perform mental tasks, the intensity of feelings of energy and fatigue, and the motivation to accomplish mental and physical tasks” (p. 697 [107]). This construct comprises three dimensions: mood (transient feeling related to energy/fatigue levels), motivation (subjective determination and enthusiasm), and cognition (sustained attention and vigilance).

The facilitative effect of glucose on sustained attention and vigilance has been demonstrated (e.g., [13,25,26,27,55]), but not consistently [26,28,35,46]. However, there is little evidence to support the facilitative effects of glucose intake on the mood and motivation dimensions of mental energy. Reay et al. [27] reported reduced mental fatigue towards the end of a cognitively demanding test battery after a 25 g glucose load. A 50 g glucose load increased subjective vigilance ratings 30 min after intake [41]. However, this was only demonstrated when participants were informed that they were consuming glucose, suggesting an expectancy, rather than metabolic, effect of energy intake. Scholey et al. [55] demonstrated that alertness ratings increased significantly after consumption of both a 25 g glucose and placebo drink contradicting any specific enhancing mood effect of glucose intake. No studies have specifically measured motivational state in relation to cognitive performance after glucose intake. Therefore, the evidence to date does not support the specific subjective mood enhancing effects of glucose intake.

### 2.4. Other Carbohydrates and Cognitive Function

#### 2.4.1. Fructose

Fructose, commonly known as fruit sugar, is a simple ketonic monosaccharide. The metabolic response profile of fructose is markedly different to that of glucose. Fructose does not significantly affect blood glucose levels, is not actively transported across the BBB, nor does it provide direct energy for cellular processes [24]. Such factors likely explain the comparative lack of research examining the effects of fructose on cognition. The available human evidence has shown facilitative effects on problem solving performance comparable to that of glucose intake (Table 3 [24]). Therefore, facilitation was evident in the presence and absence of a blood glucose response. A number of studies have highlighted that oral sensing of CHO alone is sufficient to enhance performance (e.g., [108]). This suggests a motivational, rather than metabolic, effect of CHO on performance via activation of neural reward pathways [88,91,109]. However, fructose and glucose differ in their capacity to activate motivational reward pathways; glucose activates, fructose inhibits, cortical responding [110]. Miller et al. [24] suggest activation of motivational reward pathways may not be necessary for performance facilitation. The facilitative effects of glucose and fructose may be due to activation of peripheral glucose-transport mechanisms or innervations of the vagus nerve. Increased vagus nerve activation and vagal tone have been associated with enhanced cognitive performance (e.g., [111]). However, such explanations have yet to be verified. Further, the facilitative effects of glucose may act via multiple pathways, including metabolic and peripheral mechanisms.

#### 2.4.2. Sucrose

Sucrose is a plant-derived disaccharide composed of glucose and fructose linked by an ether bond. Sucrose has the potential to affect neural function both directly, via glucose, and by indirect peripheral mechanisms, via fructose. Early studies examining the effects of sucrose in young children demonstrated no facilitative effects on cognitive performance [112,113]. A limited number of studies have directly examined the effect of sucrose on cognitive performance in adults. Attention and information processing were enhanced by 100 g of sucrose, but not 50 g glucose, in an elderly sample with mild memory complaints [114]. Sucrose may therefore proffer additional facilitative benefits to cognitive performance compared to glucose alone. Gailliot et al. [108] reported that self-control performance (suppression of homosexual stereotypes during a writing task) was bolstered by intake of a sucrose containing drink. However, very little methodological detail is provided for this study, including sucrose dose. Harte and Kanarek [115] examined the interactive effects of nicotine and sucrose intake on attention and spatial memory. Nicotine gum combined with a sucrose drink interacted to facilitate sustained attention performance compared to nicotine and an aspartame drink. Further, the sucrose drink in isolation enhanced spatial memory performance vs. the placebo. This demonstrates both the facilitative effect of sucrose and additive effects on performance when combined with nicotine. However, an enhancing effect of sucrose has not been consistently demonstrated. For example, Dye et al. [116] reported no effects of sucrose on episodic and working memory, or psychomotor function.

#### 2.4.3. Isomaltulose

Isomaltulose (Palatinose™; 6-0-α-d-glucopyranosyl-d-fructofuranose) is a naturally occurring, digestible disaccharide (C12H22O11) composed of a glucose and fructose molecule bound by a α-1,6-glycosidic bond [118]. Isomaltulose is an isomer of sucrose and is similar in taste, appearance and nutritional content but has <50% of the sweetening potential. Isomaltulose has a low glycemic value (32) which results in a slower post-consumptive rise in blood glucose and insulin production [118]. The slow absorption rate maintains raised blood glucose levels for a period of up to four hours [119]. Since isomaltulose enters the blood stream at a slower rate than sucrose, and produces lower postprandial glycemic responses [120], it is suitable for diabetics. It has also been shown to improve glycemic control in healthy men [121].

Evidence for the facilitative cognitive effects of isomaltulose is inconsistent. Isomaltulose and sucrose (40 mg) both significantly increased sustained calculation performance on a high demand task 90 min. post-consumption [117]. This performance enhancement had decreased in the sucrose, but was maintained in the isomaltulose, condition by 150 min. post-consumption. However, the two treatments were not compared statistically. Contrastingly, Dye et al. [116] found no consistent effects of an isomaltulose or sucrose milk-based drink on psychomotor performance, verbal or working memory in young healthy males.

There is modest evidence to suggest isomaltulose may proffer facilitative benefits in children. Two studies have examined the effects of adding isomaltulose to growing up milk (GUM) as a breakfast replacement or as a sweetener in a cereal based breakfast. The GUM studies were conducted in 5–6 year old children in Indonesia [122] and Malaysia [123]. Both studies documented a decline in multiple domains of cognitive performance over the morning. Isomaltulose GUM resulted in the lowest decrement in performance in a number of cognitive domains +3 h post-consumption [122,123]. However, better spatial working memory and recognition memory were found following ingestion of glucose [123]. Young and Benton [124] found no effects on cognition one hour after an equicaloric, macronutrient matched breakfast sweetened with isomaltulose or glucose in 5–11 years old children. However, improved memory and mood were observed +3 h following the isomaltulose sweetened breakfast.

### 2.5. Summary and Unanswered Questions

It is still commonly reported that 25 g of glucose is the most reliable dose for moderation of cognitive function, specifically, verbal episodic memory. However, there is sufficient evidence to suggest the ‘optimal’ dose may be dependent upon a number of mediating factors. Factors contributing to the differential susceptibility to glucose facilitation include age, task difficulty/demand, task domain, glucoregulatory control and BMI. These factors can act as direct response modifiers (e.g., task difficulty), or indirect response modifiers (e.g., glucoregulatory mechanisms, age, BMI).

Whilst there is some evidence to suggest that the memory enhancing effect of glucose follows an inverted U-shaped curve for verbal episodic memory tasks, dose–response curves may differ depending on the cognitive domain assessed. There is a distinct lack of studies that systematically vary the dose of glucose to determine the facilitative dose response effect for cognitive domains other than episodic memory. Therefore, failure to observe robust facilitation on certain cognitive tasks may simply be due to suboptimal dosing. Further research is needed to fully differentiate between the response profiles of glucose administration for different cognitive domains. Dose-response studies of other CHOs are also required.

Cognitive demand has been emphasized as a key moderator of the glucose facilitation effect, but little attempt has yet been made to define this in terms of task domain. Further examination of memory vs. non-memory tasks with variations in cognitive load is required. There are also inconsistencies in the evidence, and the proposed mechanisms of this effect are poorly explicated suggesting further examination of this moderating factor is required.

The exact role of glucoregulatory control requires further investigation due to inconsistent evidence of specific facilitation in poor and good glucoregulators. Future research should also establish which glucoregulatory index is the most efficacious predictor of the glucose effects on cognitive function. Presently, there is no consensus with regards the index of glucoregulatory control that best predicts enhanced performance in normoglycemic samples. Methods of classifying glucoregulation employed to date include fasting blood glucose levels, peak glucose levels, recovery and evoked glucose to baseline levels, and area under the curve (AUC). Implementation of the oral glucose tolerance test (OGTT) for classification purposes will help establish which glucoregulatory index is the better predictor of glucose effects on cognition. However, to date the OGTT has also been inconsistently employed (e.g., use of de-gassed Lucozade, normal Lucozade, glucose tablets dissolved in different volumes of water, timing of post ingestion capillary or venous samples, use of devices to measure these samples, period of follow-up post ingestion and analysis performed; cf. [125]).

Evidence of non-metabolic effects of glucose facilitation have been demonstrated. For example, the facilitative rewarding effect of oral rinsing, and the moderating effects of subjective expectancy and thirst. Such findings have important implications for understanding the enhancement of cognitive function by CHO intake. These effects merit further examination and at the very least should be controlled for or taken into account in the design of the dose response studies recommended.

There is limited support for the facilitative effects of non-glucose CHOs on cognitive functions but there are significant gaps in the evidence. The specific post-ingestive metabolic effects of fructose can be utilized to further examine potential non-metabolic effects of CHOs on cognitive performance. Evidence of positive effects of isomaltulose on cognitive performance is largely lacking other than in studies of potentially undernourished children in South East Asia. These samples may be more dependent on a ready supply of energy, such that any facilitative effects may be due to correcting a nutritional deficit. Thus, these studies do not provide robust indications for possible effects in well-nourished adults.

## 3. Glycemic Response and Cognitive Performance

### 3.1. Manipulating Glycaemic Response

The majority of studies investigating the effects of CHO on cognitive performance have been placebo-controlled, glucose drink interventions. A number of studies have investigated the effect of different CHOs on cognitive performance rather than just pure glucose drinks. Food interventions are typically described using terms such as glycemic index (GI), glycemic load (GL), the ratio of slowly to rapidly available glucose, the proportion of simple to complex carbohydrate, or the amount of rapidly vs. slowly digested carbohydrate. All can be considered indices of the glycemic potency of foods. The quality (e.g., type, source) and the quantity of CHO are important determinants of glycemic response. Glycemic index compares equal quantities of available CHO and thus provides a measure of CHO quality (not quantity). The GL of a food is a function of its GI and the amount of CHO per serving. Therefore, GL provided information about the quantity of CHO and reflects the glycemic response to food portions [126]. Indeed, stepwise increases in GL predict stepwise elevations in postprandial blood glucose/insulin response [127].

Glucose index reflects the rate at which an ingested substance increases and maintains blood glucose levels. High GI foods are characterized by elevated glycemic responses of short duration and a rapid return to basal levels, low GI foods typically elicit slower, more evenly sustained glycemic responses, and a slower return to basal levels over the postprandial period [126]. Therefore, food with a low GI may offer the benefit of counteracting the low blood glucose which may occur with high-GI foods in the later postprandial phase [128]. Hypoglycemia has been demonstrated to significantly impair cognitive function when induced experimentally in healthy young adults [129]. Changes in metabolite concentrations during the post-prandial period have been demonstrated to be more important determinants of cognitive performance than rather than absolute values [130,131]. This suggests the potential facilitative effects of a more balanced, steady post-prandial glycemic response, typical of low GI foods, on cognitive performance. Low GI foods induce a more moderate blood glucose peak and may maintain a prolonged net blood glucose increment above basal levels. This offers the potentially enhancing effects of maintaining adequate blood glucose availability for uptake into the brain, and additionally, may acutely improve insulin sensitivity which hypothetically offers additional benefits to postprandial performance [132].

Manipulations, that modulate glycemic and insulin response, may provide useful experimental models to examine cognitive effects. The majority of previous evidence of the enhancing effects of CHO pertains to facilitation within 1 h of an acute glucose load. A number of studies have demonstrated enhanced cognitive performance over longer postprandial periods by manipulating the GI and/or GL of food. Studies examining cognitive performance in children after breakfasts varying in GI have predominated. Modest evidence of a protective effect of low GI breakfasts on cognitive performance in children over prolonged periods of the morning has been demonstrated [133,134]. The effects of breakfasts on children’s performance may vary across cognitive domains as a function of GL and GI content [135].

Only a small number of studies have examined the effect of manipulating glycemic response on cognitive performance in the young and healthy (summarized in Table 4). The majority of this data also comes from one laboratory. Studies have focused upon breakfast manipulations and, analogous with the glucose and cognitive performance literature, have predominantly measured episodic memory. The manipulation of the rate at which glucose is made available in the blood by high and low glycemic breakfasts has resulted in facilitative effects on performance. Benton and colleagues [136,137] have shown that low GI breakfasts improve episodic memory in the late postprandial stage (150–210 min). However, no concomitant differences in blood glucose were observed in one study so the facilitative effect on cognitive outcomes cannot be attributed to late glycaemia per se. Conversely, Smith and Foster [6] reported no significant differences in episodic memory performance related to the GI of breakfasts. However, manipulation of glycemic index of breakfasts did not result in divergent blood glucose response profiles.

Individual differences in glucose tolerance may interact with glycemic load to moderate cognitive performance. For example, Nabb and Benton [138] examined the effects of eight breakfasts differing in GI, amount of CHOs and fiber. Poorer glucose tolerance resulted in more forgetting when higher levels of CHOs were consumed. Higher amounts of CHOs improved reaction time after 90 min in the poor glucose regulators. The lowest levels of fiber (1.5 g) were associated with poorer memory in subjects with poorer glucose tolerance. However, blood glucose responses were not affected by dietary fiber content, indicating that the expected variation in GI was not elicited by the composite meals, but the timing of blood glucose sampling was such that differences may have been missed. Nabb and Benton [139] also administered eight different breakfasts differing in energy content, level of CHO (24 g or 59 g), fat (1 g or 16 g) and protein (2 g or 10 g). Better glucose tolerance (categorized by fasting blood glucose levels) was associated with superior episodic memory performance. Low energy intake and low blood glucose were also associated with improved performance. Conversely, attentional vigilance and RT were enhanced in participants with good glucose tolerance and high blood glucose levels.

The conclusions from studies available to date are tempered by a range of methodological limitations (e.g., poor descriptions of meals or products ingested as well as of cognitive tests administered, insufficient standardization of the available carbohydrate content and nutrient composition of the meals, lack of adequate information on, or physiological confirmation of, the course of postprandial glycaemia, insufficient duration of the meal test and subsequent test period, or too few test subjects). The evidence to date generally favors low GI meals for improved memory and/or attention in children and elderly, and mainly in the late postprandial phase [128]. The evidence in young, healthy adults is equivocal at the present time. The beneficial effects of low GI meals may be secondary to a smoother overall blood glucose profile with sustained availability of glucose to the brain and/or to an acute improvement in insulin sensitivity. Further studies are necessitated to identify the mechanisms underpinning the facilitative effects of low GI food intake considering effects have been shown independent of divergent blood glucose response profiles. Studies of the impact of habitual consumption of low-GI vs. high-GI diets on cognitive performance are also required.

### 3.2. Moderation of Glycaemic Response by Vehicle

Manipulation of the glycemic response to foods through ingredient selection and engineering novel food structures has attracted increasing interest [140,141]. Monosaccharides and disaccharides are rapidly absorbed and elicit a rapid rise in blood glucose. Oligosaccharides (e.g., maltodextrins) and polysaccharides (e.g., starch) elicit a smaller glycemic response which may proffer benefits by maintaining the glucose response over longer timescale. For example, intake of a low GI mixture of saccharides (sucromalt) improved subjective mental energy and attenuated fatigue over 4–5 h postprandially [142]. The presence of other food constituents, such as fats and proteins, can also alter the rate of glucose absorption. These substances may, in consequence, alter the effects of glucose on cognitive performance. A number of macronutrients have potential glycemic response moderating qualities that may offer beneficial effects on postprandial glucose response profiles. The capacity of dietary fibers to reduce the overall postprandial glucose response has been demonstrated. For example, oat bran [143] and psyllium (a seed derived husk fiber [144]) regulate the rate and extent of CHOs degradation and subsequent release of glucose into the blood. Protein fractions also have significant capacity to reduce glycemic response. Milk-derived proteins are insulinogenic. Intake of 18 g of milk-derived whey protein significantly increased insulin response and lowered post-prandial glycaemia compared to white bread and controls [145,146]. A whey protein fraction has also been demonstrated to reduce glycaemia compared to a glucose reference drink in a dose-dependent manner (obese sample [147]). Gunnerud et al. [148] replicated this finding in healthy participants. Further, the insulinogenic properties of whey proteins were shown to likely be mediated by the postprandial plasma amino-acid (AA) response; whey protein affected glycaemia, insulinaemia and plasma AA response to a glucose load in a dose-dependent manner. Nine grams of whey protein was sufficient to reduce postprandial glycaemia when added to a carbohydrate-rich meal.

The potential facilitative cognitive effects of modulating the glycemic response to a glucose load by vehicle has received little attention. The available evidence offers limited support. For example, Sünram-Lea et al. [149] combined 25 g of glucose or aspartame with full fat or a fat-free yoghurt. The highest blood glucose levels were elicited by glucose combined with a fat-free yoghurt and resulted in subsequent superior short- and long-term episodic memory performance. The co-administration glucose and fat attenuated the glycemic response but no facilitative effects of glucose were demonstrated. Therefore, the slowing of glucose metabolism by fat did not result in improved performance. The authors suggest glucose may only exert its full facilitative effects if a peripheral/central facilitative glucose level is reached within a short timeframe. However, this study only examined cognitive performance up to 45 min. postprandially; facilitative effects of glycemic load manipulations may only emerge ≥150 min [136,137]. Dye et al. [116] manipulated the glycemic response with isomaltulose in a milk drink. However, no facilitative effects were observed despite an attenuation of postprandial response.

### 3.3. Summary and Unanswered Questions

The studies described above were conducted in healthy young adults using between subjects designs in which participants did not act as their own controls. The beneficial effects observed on cognitive function were apparent not at the point at which glucose levels were significantly different, but later in the post prandial period when glucose levels had returned to baseline. This could be interpreted to indicate that the metabolic challenge of a high glycemic response was more detrimental to performance even in young healthy, cognitively able, participants than the more slowly released glucose from the low GI treatment. These inferences require verification in within subjects designs with more careful control of the antecedent conditions prior to ingestion, and the use of glucose measurement that is more sensitive to change in the euglycemic range and more frequently assessed-in these studies measurements were taken usually every 30 min using devices intended to detect hyper- or hypoglycemia.

The vehicle in which ingredients selected are provided is also an important consideration. Dairy based vehicles may be insulinotrophic and modulate the glycemic response to produce a low GI profile but it is not known whether this will facilitate cognitive function and indeed only studies in nutritionally vulnerable children have demonstrated positive effects; the only study conducted in western adults did not.

There is insufficient evidence to support cognitive benefits of GL manipulations. Physiological processes other than glycaemia, such as insulinaemia, may be more closely related to changes in cognitive performance and merit systematic investigation.

## 4. Caffeine, Carbohydrates, and Cognitive Function

### 4.1. Caffeine

Caffeine is a plant and seed-derived methylxanthine that acts as a central nervous system stimulant in humans [150]. Caffeine is rapidly absorbed into the bloodstream post-ingestion via the gastrointestinal tract and can pass freely across all biological membranes, including the BBB [151]. The biological effects of caffeine are mediated by its antagonistic effects on adenosine receptors which are widely dispersed in gastrointestinal, cardiovascular, respiratory, renal, and central nervous systems [152], including the brain [153]. By inhibiting adenosine receptors, caffeine increases the release of neurotransmitters, including noradrenaline, dopamine and acetylcholine which have diverse physiological effects throughout the body (e.g., vasoconstriction in the periphery, increased blood pressure, thermogenesis, and increased renal and gastric function [154]).

The potential facilitative effects of caffeine intake on cognitive performance and psychological state have been widely examined (e.g., [155,156,157]). Broadly, performance enhancing effects have been demonstrated on psychomotor, attention, and vigilance tasks [155,158]; a less consistent effect on memory has also been reported [157,159]. Caffeine has also been consistently associated with moderation of mood, particularly increased subjective arousal, alertness and reduced mental fatigue [157,159]. The majority of this research has examined caffeine facilitation 30–60 min, after intake. Indeed, the peak maximum blood plasma concentration of caffeine is typically reached within at least an hour (e.g., [160]). The dose of caffeine commonly administered in such studies typically exceeds the natural dose present in coffee and tea (30–120 mg depending on type of bean/leaf and brewing method). For example, 250 mg of caffeine improves visual search performance, spatial selective attention and perceptual sensitivity [161,162]. However, performance enhancements have been demonstrated at lower doses ranging from 32 to 50 mg [163,164,165], and performance detriments at high doses (e.g., 400 mg [166]). A recent scientific opinion from the European Food Standards Agency (EFSA) upheld the claim that caffeine increased alertness (indexed by RT) and attention (indexed by a range of psychometric tasks) in healthy individuals of both sexes [167]. This ruling on the facilitative effects of caffeine intake was specific to doses of at least 75 mg of caffeine. A more recent ruling rejected a claim for facilitative effects of 40 mg of caffeine [168]. Whilst facilitative effects of caffeine doses <75 mg on attention and alertness performance have been demonstrated, they were considered less consistent and convincing than ≥75 mg doses. However, it is considered that this rejection of the facilitative effects of caffeine <75 mg is underpinned by the quality of the evidence to date rather than the lack capacity of caffeine to moderate cognitive performance at lower doses.

### 4.2. Combined Effects of Caffeine and CHO

#### 4.2.1. Cognitive Performance Outcomes

The facilitative effects of glucose and caffeine in isolation are well established (e.g., [60,155]). An increase in the consumption of ‘energy’ drinks, containing, amongst other ingredients, caffeine and CHOs (predominantly glucose), has intensified research interest into the potential facilitative performance and mood effects of caffeine and CHO in combination [169,170]. Table 5 summarizes studies that have examined the cognitive performance and mood effects of combined caffeine and CHO (glucose and glucose/sucrose/fructose blend) drinks, compared to glucose only, or placebo drinks (CHO- and caffeine-free). Several studies have also employed commercially available energy drinks which contain additional ingredients (e.g., taurine, glucoronolactone, and vitamin). The majority of studies have administered caffeine doses between 30 and 80 mg combined with glucose ranging between 25 and 60 g. The cognitive domains assessed have predominantly matched those established as sensitive to caffeine manipulation, namely, attention, vigilance, perceptual speed, RT, and driving performance. Relatively less attention has been given to cognitive domains shown to be sensitive to glucose intake such as episodic memory.

Significant facilitative effects of caffeine combined with CHO have been demonstrated for sustained [171,172,173] and short-term (<30 min) attention [174]. This includes event-related potential evidence (ERP; an electrophysiological measure of neural response that is considered a marker of sensory, cognitive, or motor neural events) suggesting augmented attentional information processing [172]. Kennedy and Scholey [171] propose the enhancing effects of caffeine and glucose on sustained attention may be predominantly mediated by caffeine since they demonstrated facilitative effects during the temporal period associated with peak plasma caffeine levels (+35 and +45 min. post intake). The findings of Warburton [158] also suggest that the effects of caffeine may supersede that of glucose since no effects of glucose were demonstrated when administered alone. However, since both studies did not compare combined caffeine and glucose intake with these nutrients in isolation, this proposition cannot be verified. A facilitative effect of caffeine and glucose on attention has also not been consistently reported [175].

Caffeine and CHO drinks have improved RT performance in a number of performance domains, including, behavioral control [176], visual attention [174], simple and choice RT (sleep restricted; [177]), sustained attention [172,178], and driving performance [179]. Analogous to the glucose literature, a facilitative effect of caffeine and glucose has been demonstrated under conditions of high cognitive demand. Smit et al. [178] reported enhanced RT whilst completing a fatiguing and cognitively demanding test battery. Similarly, Scholey et al. [180] demonstrated significantly faster mental arithmetic performance during a cognitively demanding multi-tasking paradigm. However, 30mg caffeine combined with 42 g sugars (glucose/fructose/sucrose blend) has been demonstrated to impair RT on a psychomotor vigilance task in sleep restricted individuals compared to a no sugar, no caffeine, sweetened control drink [181]. No effects of 80 mg caffeine and 27 g glucose/sucrose on RT has also been reported [175].

The effect of caffeine and CHO on driving performance, indexed by lane drifting, deviation of speed, and RT, has been examined. Enhanced effects have been demonstrated in the short-term (effect strongest in the first 60–90 min [179,182]) and long-term (effect evident after 3 and 4 h of prolonged driving [183]). Driver subjective sleepiness has also been examined in such studies. Intake of 80 mg:26 g caffeine:CHO was sufficient to attenuate subjective sleepiness in normal [183] and sleep restricted [182] participants. These effects were evident in the first 90 min. and the 3rd and 4th hour of sustained driving. This subjective reduced sleepiness may be specific to driving related tasks as sleepiness levels were not counteracted by intake of 30 mg:42 g caffeine:CHO during an attention vigilance task in the sleep restricted [181]. However, this effect may be mediated by the lower dose of caffeine administered in this vigilance study.

Sünram-Lea et al. [185] provide further evidence of the potential facilitative effects of caffeine and CHO in demanding contexts. Adding to evidence of performance facilitation under conditions of high cognitive demand and in the sleep deprived state, these authors reported positive cognitive effects of caffeine and CHO in individuals under conditions of stress. Activation of the psychoneuroendocrine stress response systems—the hypothalamic-pituitary-adrenal [HPA] axis and sympathetic-adrenal-medullary [SAM] system-increases the availability of metabolic glucose to cope with the demands of the stressor via the release of cortisol and adrenaline. Cortisol increases liver gluconeogenesis and decreases glucose absorption in the periphery; adrenaline increases circulating blood glucose levels via the liver. The magnitude of cortisol response to stress is moderated by glycemic status [186,187] and the release of cortisol under conditions of stress is associated with impaired cognitive function [188]. The intake of a glucose load post-stress exposure has been demonstrated to attenuate the cortisol stress response [189]. Therefore, a caffeine and glucose drink has the potential to offer performance benefits under stressful conditions. Indeed, Sünram-Lea et al. [185] report increased grip-strength and episodic memory (delayed word recall) after intake of a 40 mg:50 g caffeine:glucose drink following a fire fighting training exercise. Further, information processing was also enhanced with this dose drink and additionally with a 80 mg:12.5 g caffeine:CHO (fructose/glucose) drink.

#### 4.2.2. Subjective Outcomes

The facilitative effect of combined caffeine and CHO intake on a number of subjective state/mood indices is supported by the studies shown in Table 5. The positive effects of caffeine and glucose on ‘mental energy’ (the perception of mental alertness, high mood and motivation levels [107] has been reported [177,184]. Similarly, caffeine and glucose intake has been demonstrated to increase feelings of stimulation [176], alertness [184] and arousal [178]. Reduced mental effort during prolonged driving [183] and reduced mental fatigue [176] have also been reported. The level of cognitive demand/stress has been highlighted as a potential mediating factor in the relationship between caffeine and glucose, and subjective state. Subjective stress and anxiety after fire-fighting training exposure was attenuated by a 40 mg:50 g caffeine:glucose drink [185]. Both a 46 mg:68 g and a 33 mg:60 g caffeine:glucose drink reduced subjective fatigue during an prolonged high demand cognitive test battery [180]. These studies suggest the facilitative effects of caffeine and CHO in combination may be particularly relevant in contexts characterized by high cognitive or physical demand. The role of familiarity with the caffeine and CHO vehicle has also been highlighted [184]. Participants were exposed to a familiar (branded) and a novel energy drink (containing 30 mg:54 g caffeine:glucose) and a caffeine and CHO-free matched version of both drinks. Upon first exposure, the familiar energy drink and its branded placebo increased alertness and mental energy compared to the novel placebo suggesting an effect of familiarity with the branded drink. Facilitative effects were evident upon the second exposure only in the drinks containing caffeine and glucose; a facilitative effect of the novel caffeine and glucose drink emerging presumably as familiarity increased. 

### 4.3. Interactive Effects of Caffeine and CHO

Evidence from studies comparing the effects of caffeine and glucose combined with caffeine- and CHO-free placebo drinks presents a consistent and convincing case for the facilitative potential of these drinks across a range of cognitive domains and subjective measures of experience. However, a major limitation of the studies summarized in Table 5 is the failure to compare the combined effects of caffeine and CHO relative to the effects of these nutrients when administered in isolation. The common administration of a placebo (caffeine- and CHO-free) drink or glucose alone means it is not possible to clearly dissociate the individual and interactive effects of caffeine and CHO intake. It is therefore difficult to ascertain if the administration of caffeine in combination with CHO will proffer enhancing effects above and beyond those offered by caffeine or CHO intake in isolation. A number of the studies reported in Table 5 also administered commercial energy drinks which additionally contain potentially active agents (e.g., taurine, glucoronolactone, and vitamins) which may contribute/moderate the observed facilitative effects. Table 6 summarizes seven studies that have appropriately administered a combined caffeine and CHO dose and equivalent caffeine and glucose doses in isolation. Furthermore, a number of studies adequately controlled for additional ingredients that are commonly added to commercial energy drinks. Such designs provide some support for the facilitative effect of caffeine and CHO combined by demonstrating interactive effects of these nutrients in combination that are quantitatively or qualitatively different from the effects of caffeine or CHO administered in isolation.

#### 4.3.1. Cognitive Performance Outcomes

Two of the six studies that examined performance on attention tasks reported interactive effects of caffeine and CHO independent of the effects of these nutrients in isolation. Scholey and Kennedy [190] administered 75 mg caffeine, 37.5 g glucose and 12.5 mg of herb mix (ginseng and ginkgo biloba) in combination and isolation, as well as a placebo drink. Only the combination of ingredients improved attention speed relative to the placebo drink. Adan and Serra-Grabulosa [191] reported a facilitative effects of 75 mg:75 g caffeine:glucose on a sequential RT attentional task which was not demonstrated following intake of caffeine and glucose in isolation. Serra-Grabulosa et al. [192] reported that the same combined dose decreased neural (blood-oxygen-level dependent; BOLD) activation in areas of the prefrontal cortex associated with sustained attention processes (vs. placebo), which suggests enhanced efficiency of the attentional system. However, this effect must be treated with caution as no objective, interactive behavioral effects were demonstrated. Four studies failed to demonstrate interactive effects of caffeine and glucose (caffeine:glucose: 75 mg:75 g [192]; 200 mg:50 g [193]; 80 mg:39 g [194]; 200 mg:50 g [195]). The addition of 50g CHO (white bread) to a 200 mg caffeine capsule counteracted enhanced performance on a vigilance task compared to caffeine administered in isolation [195]. Additionally, the facilitative effects of caffeine in isolation [193], or irrespective of vehicle [194,195], on attentional performance were demonstrated.

Three of the five studies examining the effects of caffeine and CHO on memory domains reported positive interactive effects. Scholey and Kennedy [190] demonstrated that a 75 mg caffeine, 37.5 g glucose and 12.5 mg of herb mix drink improved secondary memory (composite scores across a number of immediate and delayed word and picture recall and recognition measures), but no effects on ‘speed of memory’ (composite RTs of memory tasks) or working memory. Similarly, Adan and Serra-Grabulosa et al. [191] reported an enhanced verbal learning and consolidation effect after a combined 75 mg:75 g caffeine:glucose drink not demonstrated by administration of caffeine and glucose alone. Finally, 200 mg and caffeine and 50 g glucose increased object working memory [193]. Two studies reported no interactive effects on memory (caffeine:glucose: 200 mg:37.5 g [178]; 80 mg:37.5 g [194]). Analogous to the facilitative effects on attentional performance, caffeine in isolation was also shown to enhance memory performance. For example, Giles et al. [193] reported caffeine (200 mg) to be the most consistent in the enhancement of all cognitive measures assessed, including working memory. Young and Benton [194] demonstrated that caffeine, irrespective of vehicle (yoghurt, glucose and water) enhanced memory performance (episodic and working memory).

Two studies administering 75 mg caffeine with 37.5 g glucose (composite simple and attentional RT performance [190]) and 75 g glucose (sequential RT performance [191]) reported greater RT enhancement than either substance administered alone. However, caffeine alone/irrespective of vehicle improved simple [191,193], choice [193,194], and working memory [194] RT, and was reported to be the main driver of improved simple RT [178], in a number of studies. Further, the enhancing effect of 80 mg of caffeine administered in water on RT +90 and +150 min after intake was ameliorated when this dose of caffeine was taken with glucose (37.5 g) and a yoghurt drink (3.6 glycemic load [194]). Glucose in isolation was demonstrated to both enhance simple RT and manual dexterity [191], and impair choice RT performance [193]. The selective effects of taurine were also demonstrated with impaired RT performance at low cognitive demand (simple RT) and enhanced performance at high cognitive demand (working memory RT [193]).

Controversy exists in caffeine literature regards whether the beneficial effects of caffeine intake on performance represents a genuine facilitative effect or alleviation of the impairing effects of caffeine withdrawal [196,197]. The majority of studies reviewed included a period of caffeine abstinence in the study design. Most studies adopted an abstinence period between ~6 and 24 h. Only one study specifically considered the confounding effects of caffeine withdrawal and imposed a 1 h abstinence period [173]. Positive effects of caffeine and glucose (80 mg:26 g) on attention and verbal reasoning were reported in the absence of caffeine withdrawal. James and Rogers [197] argue that many of the net effects of caffeine supplementation may be as a result of reversal of adverse withdrawal effects following short-term abstinence. Placebo-controlled studies with relatively short periods of abstinence (~1–24 h.) have predominated in the examination of the effects of caffeine and glucose. Alternative study designs may be more appropriate to examine the role of caffeine withdrawal on cognitive and subjective outcomes. Indeed, James and Rogers [197] propose that long-term withdrawal studies are the only valid method of assessing the effects of caffeine.

#### 4.3.2. Subjective Outcomes

The studies summarized in Table 6 permit clearer characterization of the capacity of caffeine and CHO combined or isolation to moderate subjective mood. Confirmatory evidence for the interactive effects of caffeine and glucose providing facilitative effects above and beyond the effects these nutrients in isolation is weak. Only Young and Benton [194] reported that the combination of 80 mg caffeine and with a low glycemic load yoghurt vehicle drink counteracted the negative mood effects of caffeine administered in water (tiredness, hostility and confusion). However, no effects of caffeine combined with a 39 g glucose load were reported suggesting a key role of glycemic load moderating the action of caffeine. Giles et al. [193] reported that adding 50 g of glucose (to 200 mg caffeine) actually potentiated caffeine-induced feelings of subjective tension. Caffeine in isolation was also shown to reduce headaches, tiredness, fatigue, and increase alertness, tension and vigor. Such effects are likely due to the elevation of caffeine withdrawal symptoms in this 24 h caffeine deprived sample. The addition of taurine to the caffeine load opposed these effects of caffeine on mood. The remaining studies that measured subjective states reported no subjective effects of caffeine and CHO intake [190,191,195].

### 4.4. Summary of Interactive Effects

The evidence for a specific synergistic effect of caffeine and CHO combined has received some support from studies examining these nutrients combined and in isolation. The facilitative effects of caffeine on attention are well known. Two studies have shown attentional facilitation, in excess of caffeine intake, by combining caffeine with glucose [190,191], and reduced activation in neural areas associated with attentional processes [192]. This suggests combined administration offers performance enhancement beyond that offered by caffeine and glucose in isolation. However, an effect of caffeine in isolation was also reported [193,194]. This inconsistency may be as a result of the discrepant attentional tasks employed. Studies demonstrating interactive effects employed a composite attentional performance score, which may be more a sensitive measure of performance [190], or placed a high level of demand on attentional resources [191]. Similarly, a specific enhancement of RT following intake of caffeine and glucose is reported for high demand tasks [190,191]. Caffeine in isolation was mostly associated with improved RT on low demand tasks (e.g., [191,193]). This suggests a specific performance facilitation effect for caffeine combined with glucose in high demand contexts. However, caffeine and glucose in combination also reduced RT [194]. The most consistent evidence for interactive effects is shown in relation to memory. Three studies have demonstrated specific facilitation of episodic [190,191] and working memory [193] only when caffeine and glucose were combined. However, specific effects of caffeine alone on memory have also been reported using the same episodic memory task and comparable caffeine:glucose dose (caffeine:glucose: 75 mg:37.5 g [190] vs. 80 mg:39 g [194]) suggesting additional, as yet unknown, moderating variables may explain the discrepancies in the evidence.

The psychostimulant effects of caffeine and CHO has been supported by evidence of increased subjective stimulation [176], alertness [184], arousal [178], mental fatigue [171,176], mental effort [183], and mental energy [177,178]. However, the majority of this evidence is from studies that did not duly administer caffeine and glucose in isolation (Table 5). To date, the evidence for interactive facilitative effects of caffeine and CHO on subjective states is weak, and in combination, may even worsen mood compared to administration in isolation.

### 4.5. Dose Effects

#### 4.5.1. Cognitive Performance Outcomes

**Figure 1:**
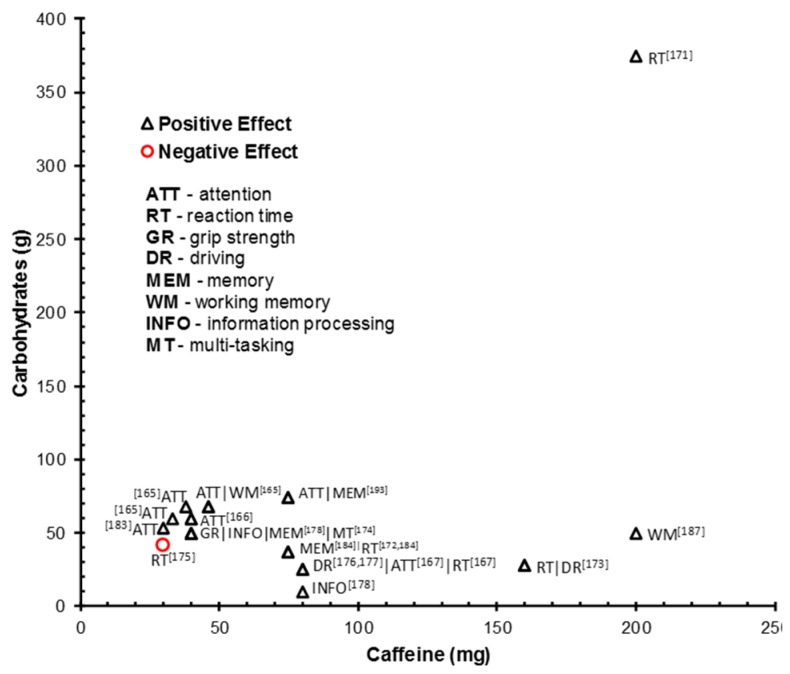
Scatterplot of cognitive performance outcomes (enhanced or impaired) by caffeine and CHO drink content. Data are representative of all studies reviewed and include multiple outcomes reported by single studies. Howard and Marczinski [176] not shown due to caffeine/CHO being administered based on body weight. Aniţei et al. [174] not shown as do not state CHO dose.

Figure 1 summarizes enhanced or impaired cognitive performance outcomes as a function of caffeine and CHO content. This is representative of all studies reviewed and includes multiple outcomes reported from single studies. The majority of the facilitative effects of caffeine and CHO on attention have been reported after intake of 33–46 mg caffeine and 54–68 g CHO. Positive attentional effects have also been demonstrated with higher caffeine:low CHO doses (80 mg:26 g caffeine:glucose and 200 mg:26 g caffeine:glucose + taurine). However, a high caffeine dose combined with a comparatively higher CHO dose (50 mg) counteracted performance facilitation of caffeine alone. In this instance, the addition of CHO removed the enhancing effect of caffeine administered in isolation. Reaction time facilitation has been demonstrated across a wider range of caffeine and CHO doses but enhanced performance is not reported below a 75 mg dose of caffeine. Indeed, RT was impaired in the sleep deprived following intake of 30 mg of caffeine (with 42 g CHO [181]). Howard and Marczinski [176] report a caffeine dose effect on behavioral control RT in which performance was enhanced following lower doses of caffeine (1.8 mg/kg = 45.6 mg for an average 78 kg participant), and diminished as caffeine dose increased (3.6 and 5.4 mg/kg). This suggests the enhancing effect of a lower caffeine dose may require specific dose calculation based on body weight. A maximal caffeine dose at which facilitation of RT occurs is not evident. For example, a 200 mg dose (combined with 375 g dextrose) enhanced simple choice RT [177]. A 275 mg caffeine dose coffee, a combined 80 mg caffeine and CHO energy drink (CHO dose not stated), and the coffee and energy drink administered together (delivering a total of 365 mg caffeine) have been shown to enhance a number of indices of RT performance [174]. However, whilst all the drinks delivered significant facilitative effects, the effects sizes were greater for the vehicles delivering the lower dose of caffeine (i.e., caffeine and energy drink in isolation).

The lowest reported dose of CHOs combined with caffeine (75 mg) to facilitate RT performance is 37.5 g. No effects on RT performance were demonstrated when a ~27 g glucose/sucrose blend was administered with a comparable dose of caffeine (80 mg [175]). This suggests a minimum CHO dose >27 g–37.5 g may be required for the enhancement of RT when combined with caffeine. A maximal dose of CHO at which facilitation of RT occurs is not evident. A 375 g dextrose dose (combined with 200 mg caffeine) has been shown to improve RT in sleep deprived individuals.

Enhanced driving performance has been demonstrated with a fairly consistent dose of CHO (26–28.25 g) combined with 80 mg and 160 mg of caffeine. However, the interactive effect of caffeine and glucose combined and in isolation has yet to be examined. Working memory has been enhanced with a high caffeine dose (200 mg) combined with 50 g of glucose [193]. No consistent moderation of working memory performance by lower doses of caffeine (75 mg) and lower and higher glucose loads (37.5 g and 75 g) suggests a dose caffeine dose effect. However, Kennedy and Scholey [171] reported a short-lived improvement in working memory performance with 46 mg:68 g caffeine:glucose. Furthermore, 80 mg dose of caffeine was sufficient to enhance working memory RT performance irrespective of whether administered in a glucose and yoghurt drinks, or water [194]. No obvious dose effect was evident for facilitation of episodic memory despite the same task being administered (immediate and delayed word recall and recognition). Both 40 mg and 75 mg of caffeine combined with (50 g and 37.5 g of glucose respectively) were sufficient to enhance performance. Conversely, no effects were demonstrated with 80 mg and 30 mg caffeine administered with 10.25–54 g of CHO. Sünram-Lea et al. [185] suggest the ratio of caffeine to glucose may be important. Only a high glucose (50 mg):low caffeine (40 mg), and not a low CHO (10.25 g fructose/glucose):high caffeine (80 mg), drink enhanced episodic memory. However, this finding may be specific to the stressful and physically demanding context employed. Hand grip strength was similarly selectively enhanced by the high glucose, low caffeine drink in this study. No such selective effects of caffeine to glucose ratio were observed for information processing which was enhanced by both ratio drinks. Further, 80 mg of caffeine alone may be sufficient to enhance episodic memory [194].

#### 4.5.2. Subjective Outcomes

**Figure 2:**
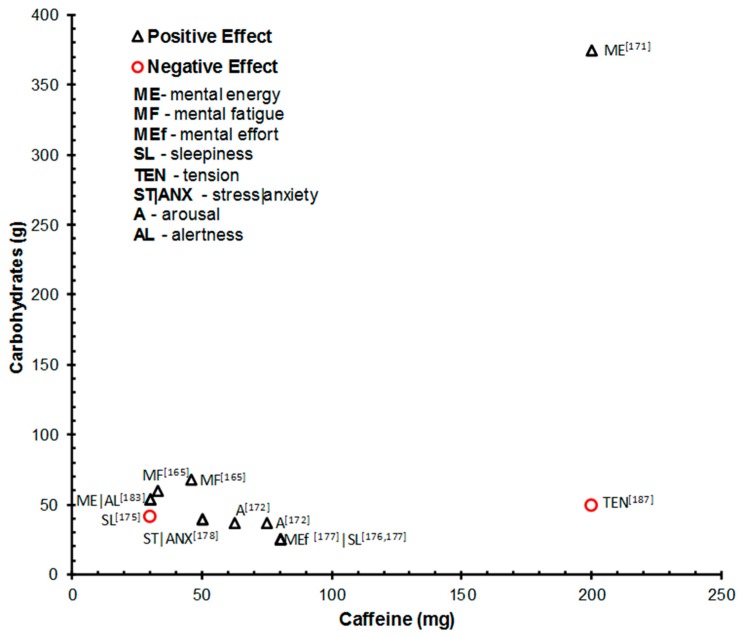
Scatterplot of subjective outcomes (enhanced or impaired) by caffeine and CHO drink content. Data are representative of all studies reviewed and include multiple outcomes reported by single studies. Howard and Marczinski [176] not shown due to caffeine/CHO being administered based on body weight. Aniţei et al. [174] not shown as do not state CHO dose.

Figure 2 summarizes enhanced or impaired subjective outcomes as a function of caffeine and CHO content. This is representative of all studies reviewed and includes multiple outcomes reported from single studies. The capacity for caffeine and CHO administration to increase energetic arousal was demonstrated with 30–200 mg of caffeine administered with comparable doses of CHO (37.5–54 g) enhancing mental energy, alertness and arousal. However, a 200 mg:50 g caffeine:glucose also drink increased subjective tension [193] or provided no additional effects than caffeine administered in isolation (200 mg capsule + bread [195]). This variability in the data may be due to the diverse and often arbitrary measures of subjective state employed. Alternatively, individual differences may result in some participants experiencing the increased arousal as positive (e.g., mental energy), and others, as negative (e.g., tension).

High caffeine (80 mg):low CHO (26 g) ratio drinks have been demonstrated to reduce subjective sleepiness and mental effort in the context of prolonged driving. This facilitation may be specific to this ratio since lower doses of caffeine (30 mg) combined double the CHO (54 g) does not counteract sleepiness in the same context. However, comparable low caffeine (33 mg and 46 mg):high glucose (68 g and 60 g) drinks reduce subjective mental fatigue. Once again, this discrepancy may more likely reflect the diverse measures of subjective experience and the variable definitions of subjective state (sleepiness vs. mental fatigue) rather than a specific dose effect per se.

The ratio of caffeine to glucose may be important in contexts of high physical and cognitive demand. Selective facilitative effects of 40 mg:50 g glucose on reduced subjective stress and anxiety have been demonstrated; facilitation was not evident after intake of a 80 mg:10.25 g caffeine:CHO drink [185].

### 4.6. Mechanisms of Action

Caffeine increases local cerebral glucose consumption [198]. Therefore, a reductive explanation is simply that the augmentation of blood glucose increases the localized effect of caffeine via increased cerebral glucose consumption. A related potential metabolic mechanism is the altered absorption and pharmacokinetic profiles of caffeine and CHO when administered concurrently. Caffeine administered in isolation increases blood glucose levels via the impairment of glucose tolerance and decreased insulin sensitivity [199,200]. The administration of caffeine with a glucose load has the potential to alter the glycemic profile of the drink. Studies that measured the post-ingestive glycemic response provide support for this proposition. Young and Benton [194] reported that 80 mg of caffeine administered with 39 g glucose or a low GL (3.6) yoghurt drink increased interstitial glucose levels, delaying peak response by 10 min and prolonging an elevated response over 90 min. post-drink, compared to these vehicles administered without caffeine. These authors also highlight the importance of considering the vehicle in which caffeine is administered and the timescale in which outcomes are assessed. No consistent effects of caffeine or glucose combined or in isolation on subjective energy levels were reported in the short term (+30 min. post intake). However, administration of caffeine alone resulted in greater tiredness in the longer term (+90 and +150 min.), whereas, administration of caffeine in a low GL yoghurt vehicle resulted in greater subjective energy over the same period.

The potential for glucose to alter caffeine absorption has also been demonstrated. Adan and Serra-Grabulosa [191] and Serra-Grabulosa et al. [192] reported that whilst caffeine alone did not moderate glucose response, salivary caffeine levels were lower when 75 mg of caffeine was administered with 75 g glucose compared to caffeine administered alone. This suggests that glucose may act to slow down the absorption of caffeine or accelerate its removal from circulation.

The action of caffeine and glucose on neurotransmitter function is another potential mechanism of the facilitative effects of these nutrients. Increased cholinergic activity has been associated with enhanced cognitive attentional and memory performance [201,202,203]. Caffeine can provoke the up-regulation of cholinergic activity via the blocking of adenosine receptors [150,204]. Further, glucose is a substrate of acetylcholine synthesis which is proposed to underpin its enhancing effects on cognitive performance [98,205]. Therefore, caffeine and glucose may proffer facilitative benefits via action on cholinergic function. Further, the effect of combining these nutrients may result in a greater increase cholinergic activity, proffering greater facilitative benefit, than that of administering caffeine and glucose in isolation. The potential moderation of neurotransmitter function by caffeine and glucose is far from limited to cholinergic activity [206]. Furthermore, the facilitative effects of caffeine and glucose are unlikely to be mediated by any one mechanism, rather by a synergy of metabolic, neurotransmitter and neuro-hormonal action [190].

Two studies reported the impairing effect of glucose and caffeine combined. Anderson and Horne [181] reported the slowing of RT (80 min post consumption) and trend for increased overall sleepiness after consuming a 30 mg:42 g caffeine:CHO drink in sleep restricted individuals. The authors emphasize that ingestion of high levels of glucose might have a short acting alerting effect, but over longer periods of time may enhance sleepiness, and thus reduce cognitive performance, in people already sleepy. This is suggestive of a specific post-prandial impairing effect of glucose, likely underpinned by rapid increase and subsequent decrease in blood glucose levels. Further, the low dose of caffeine administered may have been insufficient to counteract any fatiguing effect of glucose. However, such explanations are speculative as the authors did not compare the effect of combined caffeine and glucose with these nutrients in isolation. These findings were also only demonstrated by trend and are specific to a sleep deprived context. Giles et al. [193] did compare the combined vs. isolated effects of caffeine and glucose. Combining glucose with caffeine potentiated subjective ratings of tension. One explanation of this additive negative effect may be the augmentation of blood glucose magnified the localized effect of this high dose of caffeine (200 mg) via increased cerebral glucose consumption.

### 4.7. Unanswered Questions and Recommendations for Future Research

#### 4.7.1. Interactive Effects

More studies examining the effects of caffeine and CHO in combination and isolation are required since there are currently only seven studies to have done so. Further, only three studies report specific positive effects of combining caffeine and CHO in excess of these nutrients in isolation. Studies should also examine the different effects of dose/ratio of caffeine:CHO to evaluate the dose-dependent enhancement effects on cognitive performance and mood as well as the contribution of each nutrient in isolation. The existing evidence of facilitative effects of caffeine and CHO drinks includes studies which have examined the effects of energy drinks. The relative contribution of additional ingredients often added to these commercial caffeine and CHO drinks needs to be systematically examined. For example, taurine has been demonstrated to moderate the effects of caffeine and glucose on cognitive and subjective outcomes [193]. Further, the effects of the ingredients contained in specific energy drinks may result in multiple, systemic, and tissue-specific effects.

#### 4.7.2. Timescale of Effect

The majority of studies examining the effects of caffeine and CHO have examined outcomes during the temporal peak of caffeine and glucose responses (0–60 min). Many of the studies delayed the onset of cognitive test batteries until +30 min. to ensure adequate absorption of the combined nutrients. However, whilst plasma levels peak within an hour, caffeine has a half-life of 5–6 h in healthy adults [207]. The longer-term effects of caffeine and CHO consumption have largely been ignored. Considering evidence of the altered absorption and pharmacokinetic profiles of caffeine and CHO when administered together, facilitative, or indeed impairing, effects may be more prominent over more prolonged timescales. Further, the evidence to date is reflective of the effect of acute dosing and should not be extrapolated to the effects of chronic intake of caffeine and CHO. Whilst consistent physical performance and glucose tolerance effects are shown over chronic use [208], resistance to the physiological [209] and behavioral [210] effects following chronic intake of caffeine have been demonstrated. The proposed enhancing effect of caffeine being as a result of a reduction in the symptoms of caffeine withdrawal also necessitates longer-term study designs to confirm or refute this potential confounder in the relationship between caffeine and glucose supplementation and performance facilitation.

#### 4.7.3. Vehicle of Administration

Further research is needed to establish the effects of caffeine relative to the vehicle in which it is administered. The majority of studies have employed high GL or water-based vehicles to administer caffeine. Young and Benton [194] have demonstrated the importance of considering the vehicle/diet in which caffeine is administered and the timescale in which outcome are assessed. For example, manipulation of glycemic load of drinks can moderate subsequent effects on performance and mood over a longer timescale than the normal plasma caffeine peak. For example, traditional energy drinks combine caffeine with a high GL load (predominantly glucose). Drinks with high GL may negatively affect performance and mood 2–3 h after consumption, whereas, a low GL drinks that slowly raises blood glucose may maintain performance and mood over longer periods. Additional vehicle factors that may affect the action of caffeine and glucose include carbonation which can change the rate at which the stomach is emptied [211], thus potentially altering the rate at which nutrients are absorbed.

It is important to note that whilst caffeine can enhance cognitive performance, it is also associated with impaired glucose tolerance and insulin sensitivity [212,213], both of which have been associated with impaired cognition in the longer term.

#### 4.7.4. Effects on Subjective States

Characterization of the potential for caffeine and glucose to moderated subjective states is hampered by the diverse methods used to objectively measure subjective experience. In behavioral sciences, measures of cognitive behavior (e.g., reaction time, number of words recalled) and physiological variables (e.g., salivary cortisol concentration, blood pressure, heart rate variability) are considered to be objective measures, in contrast to, self-rating/self-reported measures of, for example, mood, which is often referred to as subjective measures. Mood can be described as a pervasive and predominant affective state and is commonly conceived to vary along orthogonal and bipolar dimensions of valence (positive vs. negative) and arousal/activation (e.g., [214,215]). Mood can also be temporally separated into protracted (e.g., depressed mood) and transient, fluctuating affective states (e.g., a momentary state of increased vigor). As mood is inherently phenomenological, it is perceived as an inconsistent measure of the brain’s output [216]. This is reflected in the common rejection of claims related to nutrients proffering benefits to subjective states, such as ‘mental energy’, which are not sufficiently characterized and have in the main not received favorable opinions from EFSA.

Some attempts have been made to clarify the validity and reliability of subjective measures of subjective state in relation to caffeine and CHO. Maridakis et al. [195] compared three measures of subjective energy and fatigue. Whilst all measures were sensitive to caffeine manipulation, some were more sensitive than others for specific aspects of subjective state (e.g., fatigue: VAS more sensitive than POMS). The inclusion of multiple measures of subjective state in nutrient manipulation studies will permit further clarification of the subjective effects of interventions and increase the construct validity of claims related to any observed changes in subjective state.

## 5. Conclusions

The potential facilitative effects of CHOs on cognitive performance have now been examined for six decades and remains a prototypical research model of the nutrition–behavior axis. The dose of glucose for which the most consistent effects on cognitive function have been observed is 25 g. These have predominantly been studies of episodic memory. Indeed, the often-quoted optimal facilitative dose of 25 g may be specific to this domain and certainly moderated by additional factors (e.g., task load, glucoregulatory control). There is a distinct lack of studies which systematically vary the dose of glucose to determine the facilitative dose response effect for cognitive domains other than episodic memory. Therefore, failure to observe robust facilitation on certain cognitive tasks may simply be due to suboptimal dosing. Further research is needed to fully differentiate between the response profiles of glucose administration for different cognitive domains. Dose-response studies of other CHOs are also required.

There is currently insufficient evidence to suggest that increasing the complexity of CHOs, or the selection of a vehicle of administration, to manipulate the glycemic response has a consistent cognitive performance benefit. Further research is needed to fully elucidate if GL manipulations offer any facilitative benefit. This should be examined across multiple cognitive domains.

Caffeine combined with glucose has been demonstrated to facilitate cognitive performance and subjective mood compared to placebo and glucose alone. However, the relative contribution of each ingredient is not ascertainable in the majority of studies. To date there have been only 7 studies which have appropriately compared the effects of caffeine and glucose alone, and in combination. Further examination of the contribution of each nutrient in isolation is required, as well as systematic manipulation of the dose/ratio of caffeine:CHO, to evaluate if there exist any dose-dependent enhancement effects on cognitive performance and mood. Studies which measure beyond the immediate absorption phase of caffeine and glucose are also needed since the half-life of caffeine is 5–6 h, and depending on the vehicle, glucose can reach the bloodstream much more rapidly (in min. rather than hours). The temporal impact of glucose and caffeine has not been clearly documented and the behavioral effects of these in dose response studies are unknown. Despite a large body of research on each constituent, the totality of the evidence is unclear since studies have not been undertaken in a systematic manner with a specific and common hypothesis. In the light of health recommendations, it is also important to consider the likely findings of longer term studies which are lacking from the literature. There have been no chronic or repeated studies of caffeine and glucose in combination and it may be important to investigate effects over the longer term particularly in the light of the evidence that caffeine increases glycemic response.

## Figures and Tables

**Table 1:** Summary of cognitive domains and associated tasks commonly employed in the literature on carbohydrate (CHO).

Cognitive Domains	Subcomponents	Cognitive Test Examples	Related Factors
Episodic Memory: Memory of autobiographical events (times, places, associated emotions, and other contextual who, what, when, where, why knowledge) that can be explicitly stated			
	Immediate Recall: (Verbal or Visual/spatial). Learning/encoding and recall of new information	Logical or Paragraph memory, List Learning tasks (e.g., California Verbal Learning), Paired Associate Verbal Learning Test; Pattern Recall	Primacy/Recency effects: Stimuli shown at the beginning (primacy) and the end (recency) of a presentation are more likely to be recalled Emotional valence: The intrinsic attractiveness (positive valence) or aversiveness (negative valence) of an event, stimuli, or situation
	Delayed Recall: (Verbal or Visual/spatial) Recall of previously learned information	As above	
	Recognition: (Verbal or Visual/spatial/faces). Ability to accurately recognize learned information (in the case of source monitoring, identifying the context in which the information was learned)	As above	
Semantic Memory: General knowledge (facts, ideas, meaning and concepts) accumulated throughout life that can be retrieved without reference to the circumstances in which it was originally acquired		Tests of general knowledge	
Implicit Memory: The use of previous experiences to aid the performance of a task without conscious awareness of these previous experiences			
	Procedural memory: Memory for performance of particular types of action. Procedural memory guides the processes we perform (e.g., driving) and most frequently resides below the level of conscious awareness	Pursuit Rotor Task; Serial Reaction Time Task; divided attention tasks	
	Priming: Exposure to a stimulus influences the responses to a subsequent stimulus	Word-stem Completion Task; Lexical Decision Task; word association tests	
Attention: The behavioral and cognitive process of selectively concentrating on a discrete aspect of information, whether deemed subjective or objective, while ignoring other perceivable information. Attention can also be considered the allocation of limited processing resources			
	Attentional Capacity: Accuracy of attention span (e.g., repeating digit sequence)	Digit Span (especially Digits Forward); Digit Symbol Substitution (DSST)	Divided attention/multi-tasking: the performance of multiple tasks concurrently to apply extra demand/load on attentional resources
	Vigilance/Focus: Sustaining attention over time to detect target stimuli, often with a demand to ignore distractors	Repeated Digits Vigilance, Continuous Performance, Bakan/Rapid Visual Information Processing (RVIP); Digit/Letter Cancellation	
	Processing/Perceptual Speed: Ability to process information and execute relevant operations within the allotted time	Trail-making Test (Part A and B); Simple/Choice Reaction time	
Executive Functions: An umbrella term for the management (regulation, control) of cognitive processes, including working memory, reasoning, task flexibility, and problem solving as well as planning and execution			
	Reasoning/Planning: Thinking with conscious intent to reach a conclusion (planning involves induction, reasoning is more deductive)	Graduate and Managerial Assessment Test of Abstract Reasoning; Tower of Hanoi	
	Inhibitory Control/Self-control: Effortful inhibition of predominant responses, emotions, thoughts, and impulses, permitting behavior to vary adaptively moment to moment	Attention-switching tests; Go/No-Go; Stroop Color and Word Test	
	Working Memory: Allows information maintained in temporary storage to be manipulated for complex cognitive operations	Paced Auditory Serial Addition Task; Serial 3s, Serial 7s; Brown Peterson Trigrams; Corsi Block Tapping	
	Problem-solving: Using generic or ad hoc methods, in an orderly manner, for finding solutions to problems	Anagram Tasks; Mathematical Problem Solving	
Language: Ability to speak or perform in an acquired language			
	Verbal Fluency: Oral production of words fitting a specified category (e.g., animals) or beginning with a specified letter	Category Fluency; Phonemic fluency	
	Verbal Reasoning: Ability to read and think about information presented and apply logic to determine whether specific conclusions can be drawn from the information	Verbal Reading-Comprehension Test	
Motor Performance: Movements and motions carried out by co-ordination of the brain, nervous system, and muscles			
	Gross motor speed: Speeded gross manual dexterity	Simple tapping task	Driving: Measures of driving performance require fine, gross and psychomotor skills
	Fine motor speed: Speeded fine manual dexterity	Grooved Pegboard	
	Psychomotor skill: The physical encoding of information, with movement and/or with activities where the gross and fine muscles are used for expressing or interpreting information or concepts	Psychomotor Vigilance Task (PVT); throwing; manipulation of objects	
Reaction Time (RT): Speed of a response (in seconds or milliseconds) to a cue, stimulus or event			
	Simple RT: Speed of response to a target (e.g., pressing a button when a cross appears)	Simple Reaction Time Test	Note: RT can be used as an index of performance on other domains of cognitive function (e.g., speed of recalling words, speed of working memory performance)
	Choice RT: Analogous to simple RT except that stimulus and response uncertainty are introduced by having multiple possible stimuli and responses	2-choice Reaction Time Test	
VisuoSpatial Function: The ability to comprehend and conceptualize visual representations and spatial relationships in learning and performing a task		Judgment of Line Orientation Test; Clock Test; Hooper Visual Organization Task	

**Table 2:** Summary of studies examining the effects of glucose on cognitive performance domains and mood.

Authors	Sample Size (Age)	Dose (Glucose)	Design (Within or Between Subjects)	Cognitive Outcomes														
				Episodic Memory	Working Memory	Attention	Recognition Memory	Visuospatial Memory	Semantic Memory	Face Recognition	Verbal Fluency	Visuospatial Functioning	Executive Functioning	Problem Solving	Implicit Memory	Self-Control	Processing Speed/RT	Mood Effects
Hall et al. 1989 [22]	12 (*M* = 20) 11 (*M* = 67.4)	50 g 50 g	Within (overnight fast) Within (overnight fast)	− **O**	**O** −			− −										
Benton, 1990 [33]	20 + 40 (*M* = 20.4 & 21.05)	25 g	Between (4 h fast)			**O**												
Azari, 1991 [29]	18 (*M* = 21)	30 g	Within (10 h fast)	−			−											
Azari, 1991 [29]	18 (*M* = 21)	100 g	Within (10 h fast)	−			−											
Benton & Owens, 1993 [30]	100 (*M* = 21.7) 53 (*M* = 21.5)	50 g 50 g (+25 g at +45 & +75 min)	Between (4 h fast) Between (4 h fast)	− −				− −										
Owens & Benton, 1994 [21]	96 (*M* = 21.2)	50 g	Between (No dietary restriction)														**O** ^3^	
Craft et al. 1994 [34]	27 (*M* = 20.8) 32 (*M* = 68.5)	50 g 50 g	Within (overnight fast) Within (overnight fast)	**O** ^3,1^ **O** ^3,1^	− −						− −		− −		− −			
Benton et al., 1994 [35]	70 + 50 (*M* = 21.5 & 21.7)	50 (+25 g at +30 min)	Between (No dietary restriction)	**O** ^3^		−							−					
Parker & Benton, 1995 [36]	100 (*M =* 20.15)	50 (+25 g at +30 min)	Between (No dietary restriction)	**O** ^2^			−											
Manning et al., 1997 [31]	24 (*M* = 18.6) 23 (*M* = 67)	50 g 50 g	Within (8 h fast) Within (8 h fast)	− **O**			− −								− −			
Foster et al., 1998 [7]	30 (M = 19.5)	25 g	Between (12 h fast)	**O** ^a^	−		−	−										
Messier et al. 1998 [37]	100 (*M* = 21.3)	10 mg/kg	Between (No dietary restriction)	−														
Messier et al. 1998 [37]	100 (*M* = 21.3)	100 mg/kg	Between (No dietary restriction)	−														
Messier et al. 1998 [37]	100 (*M* = 21.3)	300 mg/kg	Between (No dietary restriction)	**O** ^4^														
Messier et al. 1998 [37]	100 (*M* = 21.3)	500 mg/kg	Between (No dietary restriction)	−														
Messier et al. 1998 [37]	100 (*M* = 21.3)	800 mg/kg	Between (No dietary restriction)	**O** ^4^														
Messier et al. 1998 [37]	100 (*M* = 21.3)	1000 mg/kg	Between (No dietary restriction)	−														
Winder & Borrill, 1998 [32]	104 (*M* = 29.2)	50 g	Between (No dietary restriction)	−														−
Messier et al. 1999 [38]	31 (*M* = 21.3)	50 g	Within (overnight fast)	**O** ^3^														
Donohoe & Benton, 1999 [39]	67 + 69 (*M* = 21.8 & 20.2)	50 g	Between (No dietary restriction)								**O**	−	−					
Metzger, 2000 [40]	34 (*M* = 21.1)	50 g	Between (9 h fast)							**O**								
Kennedy & Scholey, 2000 [23]	20 (*M* = 20.4)	25 g	Within (overnight fast)		**O** ^b^						− ᵇ							
Green et al. 2001 [41]	26 (18-40)	50 g	Between (8 h fast)	−		**O** ^5^	**O**											Vigilance ^5^
Morris & Sarll, 2001 [42]	80 (*M* = 21.2)	50 g	Between (overnight fast)		**O** ^c^													
Scholey et al. 2001 [43]	20 (*M =* 22.7)	25 g	Between (overnight fast)	− ᵇ	**O** ᵇ						− ᵇ							
Mohanty & Flint, 2001 [19]	77 (*M* = 20.6)	50 g	Between (overnight fast)									**X** ^6^						
Mohanty & Flint, 2001 [19]	78 (*M* = 20.6)	100 mg/kg	Between (overnight fast)									**O** **X** ^6^						
Sunram-Lea et al. 2001 [17]	60 (18–28)	25 g	Between (overnight fast vs. breakfast vs. lunch)	**O** ^a^	−		**O** ^a^	**O** ^a^										
Awad et al, 2002 [44]	74 (*M* = 21)	75 g	Between (overnight fast)	**O** ^a,^ᵇ														
Scholey & Fowles, 2002 [20]	35 (*M* = 23.6)	25 g	Between (No dietary restriction)									**O**						−
Sunram-Lea et al. 2011 [16]	60 (*M* = 21)	25 g	Between (2 h fast)	**O**	−		**O**	**O**										−
Sunram-Lea et al. 2002a [18]	80 (*M* = 20)	25 g	Between (2 h fast)	**O** ^a^	**O** ^a^		**O** ^a^	**O** ^a^										−
Ford et al. 2002 [45]	20 (20–23)	25 g	Within (overnight fast)	− ^6^			− ^6^											
Flint & Turek, 2003 [46]	67 (*M* = 19.49)	10 mg/kg	Between (8 h fast)			−												
Flint & Turek, 2003 [46]	67 (*M* = 19.49)	100 mg/kg	Between (8 h fast)			**X**												
Flint & Turek, 2003 [46]	67 (*M* = 19.49)	500 mg/kg	Between (8 h fast)			−												
Flint & Turek, 2003 [46]	67 (*M* = 19.49)	50 g	Between (8 h fast)			−												
Meikle et al. 2004 ^3^ [26]	14 (*M* = 21.8)	25 g	Within (overnight fast)	**O**	−	−					−	−	−					
Meikle et al. 2004 ^3^ [26]	14 (*M* = 21.8)	50 g	Within (overnight fast)	**O**	−	−					−	−	−					
Meikle et al. 2004 ^3^ [26]	11 (*M* = 38.4)	25 g	Within (overnight fast)	**O** ᵇ	**O**	**O**					−	−	−					
Meikle et al. 2004 ^3^ [26]	11 (*M* = 38.4)	50 g	Within (overnight fast)	**O** ᵇ	**O**	**O**					−	−	−					
Meikle et al. 2005 [47]	37 + 24 (*M* = 28.5 & 18.9)	25 g	Between (overnight fast)	**O** ᵇ														
Reay et al. 2006 [27]	27 (*M* = 21.9)	25 g	Within (overnight fast)		**O** ᵇ	**O** ᵇ												Mental Fatigue
Riby et al. 2006 [48]	14 (*M* = 30.1)	25 g	Within (overnight fast)	**O** ^a^					**O** ^a^		− ^a^							
Brandt et al. 2006 [49]	40 (*M* = 22)	25 g	Between (2 h fast)				− ^6^											
Gailliot et al. 2007 [50]	62 + 73 + 18	Not stated	Between													**O**		
Masicampo & Baumeister, 2008 [51]	121	Not stated	Between													**O**		
DeWall et al. 2008 [52]	37	Not stated	Between													**O**		
Morris, 2008 [53]	72 (*M* = 22.4)	50 g	Between (No dietary restriction)	**O**									−					
Riby et al. 2008 [54]	33 (35–55)	25 g	Within (2 h fast)	−	−													
Riby et al. 2008 [54]	33 (35–55)	50 g	Within (2 h fast)	**O**	−								−					
Sunram-Lea et al. 2008 [14]	56 (*M* = 20)	25 g	Between (2 h fast)				**O**											
Scholey & Kennedy, 2009 [55]	120 (*M* = 21.6)	25 g	Between (overnight fast)	− ^a^		**O** ^a^												
Scholey et al. 2009 [56]	120 *(M* = 21.6)	25 g	Within (overnight fast)	**O** ^7^		**O** ^a^	− ^a^											
Owen et al. 2010 [57]	90 (*M* = 21)	25 g	Between (12 h fast)	−			−			−					−			
Owen et al. 2010 [57]	90 (*M* = 21)	60 g	Between (12 h fast)	**O**			**O**			−					**O**			
Brandt et al, 2010 [58]	40 (*M* = 19.1)	15 g	Between (2 h fast)				− ^6^											
Brandt et al, 2010 [58]	40 (*M* = 21)	25 g	Between (2 h fast)				− ^6,^ᵇ											
Parent et al. 2011 [59]	14 (*M* = 21.4)	50 g	Within	**O** ^8^														
Smith et al. 2011 [60]	40 (*M* = 15.5)	25 g	Between (overnight fast)	**O** ^9,^ᵇ														−
Sunram-Lea et al. 2011 [16]	30 (*M* = 20)	15 g	Between (12 h fast)	−	−		−											
Sunram-Lea et al. 2011 [16]	30 (*M* = 20)	25 g	Between (12 h fast)	**O**	−**O** ^10^		**O**											
Sunram-Lea et al. 2011 [16]	30 (*M* = 20)	50 g	Between (12 h fast)	−	−		−											
Sunram-Lea et al. 2011 [16]	30 (*M* = 20)	60 g	Between (12 h fast)	−	−		−											
Jones et al. 2012 ^11^ [25]	18 (*M* = 19)	25 g	Between (12 h fast)	**X**	**X**	**O**												**Alertness**
Brandt, 2013 [61]	60 (*M* = 19.7)	25g	Between (overnight fast)										**O** ᵇ					
Scholey et al. 2013 [62]	20 (18–35)	25 g	Between (12 h fast)	**O** ^a^														
Owen et al. 2013 [13]	24 (*M* = 20)	25 g	Mixed (12 h fast)	**O** ^3^	**O** ^12^		**O**											−
Owen et al. 2013 [13]	24 (*M* = 20)	60 g	Mixed (12 h fast)	−	**O** ^12^		**O** ^13^											−
Brown & Riby, 2013 [63]	35 (*M* = 22.19)	25 g	Between (2 h fast)	**O** ᵇ		−												
Stollery & Christian, 2013 [28]	93 (*M* = 20.7)	50 g	Between	**O** ^5^		**O**												−
Miller et al. 2013 [24]	36 (*M* = 23.25)	25 g	Between (3 h fast)											**O**				
Lange & Eggert, 2014 [64]	70 + 115 (*M* = 21.80)	Not-stated	Between													−		
Stollery & Christian, 2015 [65]	80 (*M* = 22.4)	25 g	Between	**O** ^14^														−
Brandt, 2015 [12]	40 (*M* = 19.47)	25 g	Between (overnight fast)				**O** ^a^											
Macpherson, 2015 [66]	24 (*M* = 20.6)	25 g	Within (overnight fast)				− ^a^											

**O** Significant effect;—No effect; X Impairment; ᵃ Effects under dual task paradigm; ᵇ Moderating effect of task demand; ^c^ Effect independent of glucose response; ^1^ Effect of gender; ^2^ Only for words dichotically presented to right ear; ^3^ Moderated by glycoregulatory control; ^4^ Primacy effect only; ^5^ Effect moderated by expectancy of consuming glucose Between (overnight fast); ^6^ Memory for emotionally valenced words; ^7^ Mediated by thirst; ^8^ Glucose improved recall of –ive and neutral words & augmented brain activity associated with episodic memory; ^9^ Moderating effect of trait anxiety; ^10^ Spatial working memory. ^11^ Glucose & protein improved attention & processing speed at +15 min; Protein enhanced/glucose impaired memory at +60 min; ^12^ Serial 7s & spatial working memory; ^13^ Serial 3s & spatial working memory; ^14^ Temporarily improved paired associate learning/recall when administered at encoding.

**Table 3:** Summary of studies examining the effects of fructose, sucrose and isomaltulose on cognitive performance domains.

CHO Source	Authors	Sample Size (Age)	Drink (Volume/Vehicle)	Design (Within or Between Subjects)	Cognitive Outcomes											
					Verbal Episodic Memory	Working Memory	Attention	Recognition Memory	Problem Solving	Semantic Memory	Face Recognition	Verbal Fluency	Visuospatial Functioning	Executive Functioning	Psychomotor Function	Self-control
Fructose	Miller et al. 2013 [24]	36 (*M* = 23.25)	(300 mL) 25 g glucose vs. 25 g fructose vs. sucralose placebo	Between (3 h fast)					**O**							
Sucrose	Kashimura et al. 2003 [117]	14 (*M* = 40.2)	(200 mL) 40 g sucrose vs. 40 g Palatinose	Between (12 h fast)			**O**									
	Harte & Kanarek, 2004 [115]	14 (18–20)	(227.3 mL) Lemonade (17 g sucrose) vs. aspartame placebo	Within (2 h fast)			**O** ¹						**O**			
	Gailliot et al. 2009 [108]	56	(397.7 mL) Sucrose vs. sucralose	Between												**O** ²
	Dye et al. 2010 [116]	24 (18–32)	(429 mL) Milk-based drink containing isomaltulose vs. sucrose vs. water	Within (overnight fast)	−	−									−	
Isomaltulose	Kashimura et al. 2003 [117]	14 (*M* = 40.2)	(200 mL) 40 g sucrose vs. 40 g Palatinose	Between (12 h fast)			**O**									
	Kashimura et al. 2003 [117]	14 (*M* = 32.8)	(185 g) 5 g Palatinose vs. (180 g) 10 g Palatinose	Between (12 h fast)			**O**									
	Dye et al. 2010 [116]	24 (18–32)	(429 mL) Milk-based drink containing isomaltulose vs. sucrose vs. water	Within (overnight fast)	−	−									−	

**O** Significant effect;—No effect; X Impairment; ^1^ Interactive, additive effects when combined with nicotine gum; ^2^ Reduced stereotyping and prejudice attitudes.

**Table 4:** Summary of studies examining the effects of manipulating glycemic response on cognitive performance domains.

Authors	Sample Size (Age)	Intervention	Design (Within or Between Subjects)	Cognitive Outcomes						
				Verbal Episodic Memory	Executive Function	Working Memory	Attention	Processing Speed/RT	Problem Solving	Moderating Effect of Postprandial Glycemic Response
Benton et al. 2003 [137]	71 (*M* = 21)	High-SAG biscuit, 50 g: 34 g CHO (8 g SAG + 20 g RAG, GI = 42) vs. Low-SAG cereal bar, 50 g: 31 g CHO (0.05 g SAG + 21 g RAG, GI = 66)	Between (overnight fast)	**O**						Enhanced after low GI at 150 & 210 min
Benton & Nabb 2004 [136]	323 (*M* = 21)	No breakfast vs. High-SAG biscuit, 50 g: 34 g CHO (7.9 g SAG, 18.8 g RAG, GI = 42) vs. Low-SAG cereal bar, 49 g: 34 g CHO (0.4 g SAG + 21.6 g RAG, GI = 66) or (0.05 g SAG + 21.10 g RAG)	Between (overnight fast)	**O**			−			Enhanced after low GI at 210 min
Nabb & Benton, 2006b [139]	189 (*M* = 20)	8 breakfast conditions differing in energy (114–407 kcal), & contained either low or high levels of CHO (24 or 59 g), fat (1 or 16 g) or proteins (2 or 10 g)	Between (overnight fast)	**O**			**O**	**O**		Episodic: better glucose tolerance, low caloric intake & lower levels of blood glucose = enhanced performance. RT & vigilance: better glucose tolerance, higher levels of blood glucose = faster RT and better vigilance
Nabb & Benton, 2006a [138]	168 (*M* = 20)	8 breakfast conditions differing in contents of available CHO and dietary fiber: Low carb (15 g) with low or medium DF [100 mL milk vs. Medium CHO (30 g) with low, medium or high DF [200 mL milk] vs. High CHO (50 g) with low, medium or high DF [200 mL milk]	Between	**O**						Episodic: high carb meal + better glucose tolerance = forgot less words vs. poor glucose tolerance ppts. Poor glucose tolerance + low carb meal = forgot less words vs. high carb meal & poorer word recall after low vs. high fiber. Attention: better glucose tolerance + medium and high carb meals = faster RT (90 min)
Smith & Foster, 2008 [6]	36 (*M* = 15.6)	30 g All-Bran (GI = 30) vs. 30 g Cornflakes (GI = 77). Served with 125 mL of milk	Between (overnight fast)	− ^a^ **O** ^a^						Episodic: no effect on verbal learning. High GI = fewer items forgotten in long delay recall vs. short delay (vs. low GI)
Micha et al. 2010 [135]	60 (*M* = 13)	Classification of habitual breakfast intake into 4 groups: HIGH GL:low or high GI and LOW GL:low or high GI	Between (overnight fast)	**O** ^a,1^ − ^b^	−	**O** ^2^		**O** ^2^	**O** ^3^	Fractionation of effects on specific cognitive tests by GL and GI breakfast forms. Enhancing effects in High GL forms which were associated with higher BG levels ~120 min post ingestion

**O** Significant effect;—No effect; X Impairment GL—glycemic load; GI—glycemic index; SAG—slowly available glucose; RAG—rapidly available glucose; CHO—carbohydrate; DF—dietary fiber; ᵃ Effects under dual task paradigm; ^b^ Immediate word recall; ^1^ High GI breakfast only; ^2^ Low-GI, high-GL breakfast only; ^3^ High-GL breakfast only.

**Table 5:** Summary of studies examining the effects of caffeine and CHO in combination on cognitive performance and subjective mood.

Author	Sample Size (Age)	Design (Within or Between Subjects)	Performance Measured (Relative to Drink Intake)	Drink (Volume/Vehicle)	Outcome Measures	Outcomes
Horne & Reyner, 2001 [179]	11 (*M* = 24)	Within (restricted sleep (5 h); overnight caffeine fast)	30 min drive–30 min break (drink)–2 h driving	(500 mL) caffeine 160 mg + 28.25 g CHO (11.3 g/100 mL) vs. placebo energy drink	Driving simulator (lane drifting and RT)	Caffeine + CHO significantly improved both lane drifting and RT. Effect strongest in 1st h
Warburton et al. 2001 [173]	Study 1: 20; Study 2: 22 (18–24)	Within (1 h caffeine abstinence)	+45 min	(250 mL) (Red Bull) 80 mg caffeine + 21 g sucrose + 5 g glucose +1 g taurine vs. Study 1: sugar-free water; Study 2: water + ~6 g glucose	RVIP; verbal reasoning; verbal and non-verbal memory test; Bond-Lader mood VAS	Energy drink improved attention, and verbal reasoning RT vs. glucose and non-glucose placebo, and reduced variability in RT performance. No difference between glucose and non-glucose drinks. No memory effects
Reyner & Horne, 2002 [182]	12 (*M* = 24)	Within (overnight caffeine fast; restricted sleep (5 h))	30 min drive–30 min break (drink)–2 h driving	(250 mL) (Red Bull) 80 mg caffeine + 21 g sucrose + 5 g glucose vs. placebo version	Driving simulator (lane drifting and RT); EEG; Karolinska Sleepiness Scale	Caffeine + CHO = reduced sleep-related driving incidents and subjective sleepiness during the afternoon. Effect strongest in 1st 90 min
Kennedy & Scholey, 2004 [171]	Study 1: 30 (18–25); Study 2: 26 (18–24)	Double-blind, placebo-controlled, cross-over design (24 h; overnight fast and caffeine abstinence)	+10 min	Study 1: (380-mL) 38 mg caffeine + 68 g glucose vs. 46 mg caffeine + 68 g of glucose, vs. vehicle placebo; Study 2: (330-mL) 33 mg caffeine + 60 g glucose vs. just the vehicle.	10 min cognitive test battery × 6 times (=60 min cog. demand): Serial 3s and 7s; RVIP; mental fatigue VAS	Both studies: improved accuracy of RVIP performance with all 3 active treatments. Effects emerged + 35 (38g and 46g caffeine) and +45 (33g caffeine) min after drink intake. 46 mg caffeine drink improve WM in initial 2 blocks. Higher dose of caffeine (46 mg) and caffeine drink (33 mg) reduced self-assessed mental fatigue during the extended period of cognitive performance (no effect of 38 g = baseline effect?)
Smit et al. 2004 [178]	Study 1: 28 (18–49); Study 3: 97 (18–55)	Study 1: Within (overnight caffeine abstinence); Study 3: Between (CHO (breakfast) deprived)	+5–+90 min	(250 mL) Study 1: 75 mg caffeine + 37.5 g glucose vs. placebo vs. water; Study 3: 62.5 mg caffeine + 37.5 g glucose vs. 62.5 mg caffeine vs. 62.5 mg caffeine + 37.5 g glucose non-carbonated	Simple RT; RVIP; immediate and delayed word recall; letter search task; mood VAS	Caffeine + glucose drinks improved and/or maintained mood (arousal) and RT performance during fatiguing and cognitively demanding tasks relative to placebo
Rao et al. 2005 [172]	40 (18–30)	Between (no fasting; caffeine abstinence on test day)	Not known	(330 mL) 40 mg caffeine + 60 g glucose syrup vs. sweetness/flavor matched placebo	BP; HR; EEG; ERP; sustained selective attention	Glucose + caffeine drink = improved accuracy and RT on sustained selective-attention task vs. placebo. Glucose + caffeine = improved stimulus processing at several stages of information processing (ERP)
Anderson & Horne, 2006 [181]	10 (=22.4)	Double blind, crossover design (1 week; restricted sleep (5 h); taken with soup lunch; ~14 h caffeine abstinence)	+10 min	(250 mL) 30 mg caffeine + 42 g sugars (glucose, fructose, sucrose) vs. sugar- caffeine-free orange flavored drink	Psychomotor Vigilance Test; Karolinska Sleepiness Scale	Energy drink did not counteract sleepiness and = slower RTs and more lapses 80 min post-intake
Smit et al. 2006 [184]	76 (18–40)	Between (overnight food and caffeine fast)	+7–+120 min	(330 mL) Familiar drink: 30 mg caffeine + 54 g glucose vs. familiar drink placebo vs. Novel drink: 30 mg caffeine + 54 g glucose vs. novel drink placebo	Simple RT; RVIP; serial 7’s; letter search task; mood VAS	First exposure: familiar drink and its placebo improved alertness, mental energy and mental performance vs. baseline and novel placebo drink. Repeated exposure/increased familiarity with the novel drinks: both caffeine + CHO containing drinks = sustained beneficial effects vs. placebo drinks and baseline measures
Childs & de Wit, 2008 [177]	35 (18–35)	Within (caffeine abstinence on test day)	Remained awake 5 p.m.–5 a.m. Energy capsule or placebo 3:30 a.m. Cog. testing +30 min	(Capsule) 200 mg caffeine + 50 mg white willow bark + 30 mg magnesium oxide + 10 mg taurine + 375 g dextrose vs. 375 g dextrose placebo	BP; physical activity meter; Simple and choice RT task; POMS and mood VAS	Caffeine = improved mood and mental energy and counteracted increases in simple and choice RT vs. placebo
Gendle et al. 2009 [175]	36 (18–21)	Within (4 h fast and caffeine abstinence)	+30 min	(250 mL) 80 mg caffeine + 1000 mg taurine + 27 g glucose/sucrose vs. sugar and caffeine free version	Visual attention and RT (Conner’s Continuous Performance Test II)	No effects
Howard & Marczinski, 2010 [176]	80 (*M* = 20.1)	Between (2 h fast; 8 h caffeine abstinence)	+30 min	Energy drink doses calculated by body weight. Caffeine content for average 78 kg ppt given in (): 1.8 mL/kg energy drink (45.6 mg) vs. 3.6 mL/kg energy drink (91.2 mg/30.8 g CHO) vs. 5.4 mL/kg energy drink (136.7 mg) vs. 3.6 mL/kg placebo drink (29.3 g CHO) vs. no drink)	Cued go/no-go task; mental fatigue VAS	Energy drink = increased stimulation, decreased mental fatigue, and decrease behavioral control RT. No effect on response inhibition. Lowest caffeine dose = greater RT and subjective measure improvement. Improvements diminished as the dose increased
Mets et al. 2011 [183]	24 (*M* = 21–35)	Within	Drive 2 h–drink intake–drive 2h	(250 mL) (Red Bull) 80 mg caffeine + 21 g sucrose + 5 g glucose + 1 g taurine + vs. placebo (Red bull) drink	STISIM Drive™ driving simulator (standard deviation of lateral position (SDLP); standard deviation of speed); subjective driving quality and mental effort; Karolinska Sleepiness Scale	Energy drink significantly improved driving relative to placebo: SDLP reduced in 3rd and 4th h. Reduced standard deviation of speed, improved subjective driving quality, and reduced mental effort during 3rd hr. Subjective sleepiness was significantly decreased in 3rd and 4th h of driving
Aniţei et al. 2011 [174]	153 (18–21)	Between	+40 min	275 mg caffeine coffee vs. energy drink (1000 mg taurine + 80 mg caffeine + sucrose/glucose (not stated) vs. 275 mg caffeine + energy drink vs. no drink	Perceptual speed; visual and auditory attention RT; visual orientation performance; vigilance test	Caffeine alone and combined with CHO in energy drink increased motor reactivity, short-term attention (under 30 min) and visual attention RT. Effects less consistent/smaller when caffeine and energy drink combined (365 mg caffeine)
Sünram-Lea et al. 2012 [185]	81 (*M* = 26)	Between (overnight fast + standardized breakfast; caffeine abstinence from waking)	+10 (pre-stressor) and +60 min (post-stressor)	(330-mL) 40 mg caffeine + 50 g glucose vs. 80 mg caffeine + 10.25 g fructose (41%)/glucose (59%) vs. placebo drink	Salivary cortisol; CBG; immediate and delayed free word call; letter cancellation task; grammatical reasoning task; letter digit substitution task; hand grip strength	50 g glucose +40 mg caffeine =increased grip strength and improved memory performance. Both active drinks = improved information processing (letter-digit substitution task) performance vs. placebo. 50 g glucose/40 mg caffeine = reduced anxiety and subjective stress. No effects on reasoning and attention or subjective alertness
Scholey et al. 2014 [180]	150 (18–55)	Between (12 h fast and caffeine abstinence)	+30 min	(330 mL) 40 mg caffeine + 60 g glucose vs. 25 g glucose vs. 60 g glucose	CBG; salivary caffeine level; multi-tasking framework (4 simultaneous tasks: mathematical processing task; stroop; memory search; target tracker task); Bond–Lader mood VAS; stress and fatigue VAS	Co-administration of glucose and caffeine = greater multi-tasking performance than placebo or glucose alone

CHO—carbohydrate; EEG—electroencephalogram; VAS—visual analogue scale; RVIP—Rapid Visual Information Processing. EEG—electroencephalogram; VAS—visual analogue scale; RVIP—Rapid Visual Information Processing; ERP—event-related potential; BP—blood pressure; HR—heart rate; POMS—Profile of Mood States. CHO – carbohydrate; VAS—visual analogue scale; SDLP—standard deviation of lateral position. VAS—visual analogue scale; CBG—capillary blood glucose.

**Table 6:** Summary of studies examining the effects of caffeine and CHO in combination and isolation on cognitive performance and subjective mood.

Author	Sample Size (Age)	Design (Within or Between Subjects)	Performance Measured (Relative to Drink Intake)	Drink [Volume/Vehicle]	Outcome Measures	Outcomes	Interactive Effect of Caffeine and Glucose
Smit et al. 2004 [178]	Study 2: 146 (18–54)	Between (overnight caffeine abstinence)	+5–+90 min	75 mg caffeine + 37.5 g glucose vs. 37.5 g glucose vs. 75 mg caffeine vs. 75 mg caffeine + 37.5 g glucose non-carbonated	Simple RT; RVIP; immediate and delayed word recall; letter search task; mood VAS	Main treatment effect suggesting caffeine = main component associated with improved simple RT and increased arousal; comparatively minor, weak effects of CHO demonstrated	**x**
Scholey & Kennedy, 2004 [190]	20 (18–32)	Within (overnight fast; morning coffee abstinence)	+30 min	(250 mL) Placebo (artificially flavored and sweetened water vehicle) vs. vehicle + 75 mg caffeine vs. vehicle + 37.5 g glucose vs. vehicle + flavoring levels of herbs (12.5 mg ginseng extract and 2.004 mg ginkgo biloba extract) vs. complete energy drink (75 mg caffeine, 37.5 g glucose + flavoring levels of herbs)	CBG; HR; Digit Symbol Substitution Task; CDR (immediate and delayed word + picture recall and recognition; Simple and choice RT; digit vigilance; spatial and numeric WM. Factor analyzed for global “quality of memory” outcomes; Bond-Lader mood VAS; POMS	No effect of glucose/caffeine/herbs in isolation. Whole drink = improved “Secondary memory” (combined % accuracy scores delayed word recognition, delayed picture recognition, immediate word recall and delayed word recall) and “speed of attention” performance vs. placebo (only)	**√**
Maridakis et al. 2009 [195]	17 (*M* = 23.8)	Within (8 h fast)	~+30 min	(Capsule) 200 mg caffeine + 50 g CHO (white bread) vs. 200 mg caffeine vs. 50 g CHO vs. placebo capsule vs. 50 g CHO + placebo pill	CPT; BAKAN; POMS; Activation-Deactivation Checklist; State-Trait Energy and Fatigue scales	Caffeine improved attention. No additional performance benefit of adding CHO. Caffeine increase energy, lowered fatigue. No additional benefit of adding CHO. CHO in isolation = less effects on mood	**x**
Adan & Serra-Grabulosa, 2010 [191]	72 (18–25)	Between (8 h fast; 18 h caffeine abstinence)	+30 min	(150 mL) water vs. water + 75 mg caffeine vs. water + 75 g glucose vs. water + 75 mg caffeine/75 g glucose	CBG; salivary caffeine level; RAVLT; Purdue-Pegboard; Benton Judgement of Line Orientation Test (visuo-spatial function); CCAP (attention, RT and visual scanning speed); digit span; mood VAS	Caffeine + glucose = beneficial effects on attention (sequential RT tasks) and verbal memory learning and consolidation (not shown by ingredients in isolation). Caffeine alone = improved simple RT. Glucose alone = improved simple and sequential RT tasks and manual dexterity assembly task.	**√**
Serra-Grabulosa et al. 2010 [192]	40 (18–25)	Between (8 h fast; 12 h caffeine abstinence)	+30 min	(150 mL) Water + 75 g glucose vs. water + 75 mg caffeine vs. water + 75 g glucose/75 mg caffeine	CBG; salivary caffeine level; CPT (sustained attention); fMRI	No effect of drink on cognitive performance. Glucose + caffeine = decreased activation in the bilateral parietal and left prefrontal cortex (areas associated with sustained attention and WM processes). Interpreted as increased efficiency of the attentional system	**√**
Giles et al. 2012 [193]	48 (*M* = 20.08)	Mixed (standardized meal +2 h fast; 24 h caffeine abstinence)	+30 min (WM); +60 min (RT)	(Capsule) Within (all P’s): 200 mg caffeine/0 mg taurine vs. 0 mg caffeine/2000 mg taurine vs. 200 mg caffeine/2000 mg taurine vs. 0 mg caffeine/0 mg taurine; [500 mL] Between (50:50 sample split) 50 g glucose vs. 50 g stevia	HR; Attention network test (alerting, orienting, executive control); N-back task; simple and choice RT; salivary cortisol; POMS	Caffeine = most consistent effects on cognitive performance. Glucose slowed RT. Glucose + caffeine enhanced object WM. Glucose + taurine, enhanced orienting attention. Taurine = selective effects (+ive at high load). Caffeine reduced headache symptoms and tiredness and increased alertness. Caffeine reduced fatigue and increased feelings of tension and vigor. Glucose potentiated caffeine-induced feelings of tension. Taurine intake opposed caffeine effects on mood	**x**
Young & Benton 2013 [194]	345 (*M* = 21.78)	Between (2 h fast)	+30; +90; +150 min	(250 mL) yoghurt (GL = 3.6) + no caffeine vs. yoghurt (GL = 3.6) + 80 mg caffeine vs. 39 g glucose (GL = 30) + no caffeine vs. 39 g glucose (GL = 30) + 80 mg caffeine vs. flavored water + no caffeine vs. flavored water + 80 mg caffeine	CBG and CGMS (subsample *n* = 38); immediate and delayed word recall; choice RT; serial sevens; arrow flankers (selective attention); vigilance/sustained attention; POMS	Caffeine, irrespective of vehicle, = better memory, faster RT (choice reaction time test and WM) and increased vigilance. Greater subjective energy reported 30 min after consuming caffeine and water, vs. water alone; after 90 and 150 min caffeine administered in water increased tiredness, hostility and confusion. Combining caffeine with a yoghurt-based drink increased energy, agreeableness and clear-headedness later in the morning. No effects of caffeine + glucose on mood	**x**

**x**—effect; **√**—no effect; GL—glycemic load; WM—working memory; CBG—capillary blood glucose; HR—heart rate; POMS—Profile of Mood States; CGMS—continuous glucose monitoring system; CPT—continuous performance task.
